# Transplantation of human neural progenitor cells secreting GDNF into the spinal cord of patients with ALS: a phase 1/2a trial

**DOI:** 10.1038/s41591-022-01956-3

**Published:** 2022-09-05

**Authors:** Robert H. Baloh, J. Patrick Johnson, Pablo Avalos, Peggy Allred, Soshana Svendsen, Genevieve Gowing, Kristina Roxas, Amanda Wu, Becky Donahue, Sheryl Osborne, George Lawless, Brandon Shelley, Koral Wheeler, Carolyn Prina, Dana Fine, Tami Kendra-Romito, Haniah Stokes, Vicki Manoukian, Abirami Muthukumaran, Leslie Garcia, Maria G. Bañuelos, Marlesa Godoy, Catherine Bresee, Hong Yu, Doniel Drazin, Lindsey Ross, Robert Naruse, Harish Babu, Eric A. Macklin, Ashley Vo, Ashraf Elsayegh, Warren Tourtellotte, Marcel Maya, Matthew Burford, Frank Diaz, Chirag G. Patil, Richard A. Lewis, Clive N. Svendsen

**Affiliations:** 1 Board of Governors Regenerative Medicine Institute, Cedars-Sinai Medical Center, Los Angeles, CA USA; 2 Department of Neurology, Cedars-Sinai Medical Center, Los Angeles, CA USA; 3 Cedars-Sinai Spine Center, Los Angeles, CA USA; 4 Biostatistics Core, Cedars-Sinai Medical Center, Los Angeles, CA USA; 5 Department of Neurology, Massachusetts General Hospital, Boston, MA USA; 6 Department of Neurosurgery, Cedars-Sinai Medical Center, Los Angeles, CA USA; 7 Department of Anesthesiology, Cedars-Sinai Medical Center, Los Angeles, CA USA; 8 Biostatistics Center, Massachusetts General Hospital, Harvard Medical School, Boston, MA USA; 9 Cedars-Sinai Comprehensive Transplant Center, Los Angeles, CA USA; 10 Department of Pulmonary and Critical Care, Cedars-Sinai Medical Center, Los Angeles, CA USA; 11 Department of Pathology and Laboratory Medicine, Cedars-Sinai Medical Center, Los Angeles, CA USA; 12 Department of Imaging, Cedars-Sinai Medical Center, Los Angeles, CA USA; 13 Present Address: Novartis Institutes for BioMedical Research, Cambridge, MA USA; 14 Present Address: Fujifilm Cellular Dynamics, Inc., Madison, WI USA; 15 Present Address: USC Mark and Mary Stevens Neuroimaging and Informatics Institute, Los Angeles, CA USA; 16 Present Address: Department of Psychiatry and Biobehavioral Sciences, UCLA, Los Angeles, CA USA; 17 Present Address: Department of Neurosurgery, Providence Regional Medical Center Everett, Everett, WA USA; 18 Present Address: Department of Neurosurgery, SUNY-Upstate Medical University, Syracuse, NY USA

**Keywords:** Amyotrophic lateral sclerosis, Amyotrophic lateral sclerosis

## Abstract

Amyotrophic lateral sclerosis (ALS) involves progressive motor neuron loss, leading to paralysis and death typically within 3–5 years of diagnosis. Dysfunctional astrocytes may contribute to disease and glial cell line-derived neurotrophic factor (GDNF) can be protective. Here we show that human neural progenitor cells transduced with GDNF (CNS10-NPC-GDNF) differentiated to astrocytes protected spinal motor neurons and were safe in animal models. CNS10-NPC-GDNF were transplanted unilaterally into the lumbar spinal cord of 18 ALS participants in a phase 1/2a study (NCT02943850). The primary endpoint of safety at 1 year was met, with no negative effect of the transplant on motor function in the treated leg compared with the untreated leg. Tissue analysis of 13 participants who died of disease progression showed graft survival and GDNF production. Benign neuromas near delivery sites were common incidental findings at post-mortem. This study shows that one administration of engineered neural progenitors can provide new support cells and GDNF delivery to the ALS patient spinal cord for up to 42 months post-transplantation.

## Abstract (web summary)

A phase 1/2a study shows that human neural progenitor cells modified to release the growth factor GDNF are safely transplanted into the spinal cord of patients with ALS, with cell survival and GDNF production for over 3 years.

## Main

Approximately 5,000 cases of ALS arise in the USA annually. Symptoms result primarily from the death of spinal and cortical motor neurons, resulting in paralysis and death typically within 3–5 years of onset. Riluzole and edaravone, the only treatments approved by the US Food and Drug Administration (FDA), only modestly slow disease progression^1^. Gene mutations underlie ~10% of ALS cases^2^, with gene-targeted therapies under development^3,4^. But the cause of sporadic ALS remains a mystery.

Although embryonic stem cells can generate motor neurons in culture^5^, re-forming appropriate connections in vivo in adults following transplantation is challenging given the need for long peripheral projections to muscles. Adult stem cells have not provided sustained functional improvement in patients with ALS to date^6^. An early phase 2 trial using adult bone marrow-derived mesenchymal stem cells (MSCs) suggested a transient slowing of disease progression, presumably through MSC production of trophic factors and anti-inflammatory properties^7^. A separate phase 2 trial led by Brainstorm showed promising efficacy data with MSCs^8^; however, the larger phase 3 trial failed to reach clinical significance (NCT03280056). Several trials (NCT01348451; NCT01730716) have injected fetal human spinal cord-derived neural stem cells (termed NSI-566) into the spinal cord of patients with ALS to provide new interneurons and perhaps growth factor release to protect motor neurons^9–11^. Some grafts survived but had no impact on disease. However, these trials showed the safety of cell injections into the human ALS spinal cord, a critical outcome for future similar approaches.

Astrocytes, known to support neuronal function, may be defective in ALS and contribute to motor neuron death^12^. It has been shown that healthy astrocytes can protect diseased motor neurons^13^. Therefore, restoring healthy astrocytes through transplantation may mitigate motor neuron damage and slow ALS disease progression^14^. In decades of preclinical studies, we have transplanted cortical-derived human neural progenitor cells (hNPCs) into the rodent, pig and nonhuman primate central nervous system (CNS) where cells survive, differentiate into astrocytes, do not form tumors and can protect host cells and function in models of degenerative disease and aging^15–28^. However, wild-type hNPCs transplanted into the lumbar spinal cord of a superoxide dismutase 1 (SOD1) transgenic rat model of ALS did not slow motor neuron death or disease progression^16,25^; this is supported by other groups^29^ and suggests that further trophic support may be required.

GDNF is a potent growth factor for dopamine and motor neurons; however, it cannot cross the blood–brain barrier^30,31^. Clinical trials for Parkinson’s disease confirmed the safety of GDNF intraparenchymal delivery^32,33^. However, in ALS trials with subcutaneous and intrathecal administration, GDNF had a short plasma life and low penetration into the brain and spinal cord, resulting in poor access to motor neurons and a lack of effect^34^. This led us to combine astrocyte replacement and GDNF delivery direct to the spinal cord for a cell and ex vivo gene therapy approach to protect dying motor neurons. Unlike hNPCs alone, transplanted hNPCs genetically engineered to stably produce GDNF could preserve motor neurons in the SOD1 rat lumbar spinal cord^16,25^. We have confirmed that these cells have long-term survival without tumor formation and can be safely delivered to the aged spinal cord, where they also differentiate into astrocytes and protect motor neurons^26,27^.

A single human fetal cortical sample was used to derive a neural progenitor cell line, termed CNS10, subsequently transduced for stable GDNF expression^35^. These cells were expanded and banked under Good Manufacturing Practice (GMP) to generate the product CNS10-NPC-GDNF. Here, we outline the investigational new drug (IND)-enabling preclinical studies in rodents and pigs, and findings from the first combined stem cell and ex vivo gene therapy trial for ALS. This 12-month phase 1/2a dose-escalation trial, which transplanted CNS10-NPC-GDNF unilaterally into the lumbar spinal cord of 18 patients with ALS, met the safety endpoint. The transplant had no negative effect on motor function, and sustained graft survival and GDNF production were observed in all post-mortem tissue. The transition zone of the spinal cord was targeted, with the prediction that cells would migrate to the ventral horn. However, most transplants were in the dorsal horn, possibly contributing to a lack of overall motor neuron protection and to pain in some participants. Graft location, along with GDNF secretion, may have also contributed to the instances of neuromas containing Schwann cells at the dorsal root entry zone (DREZ). However, overall, this study demonstrates that this combined cell and gene therapy can safely deliver astrocytes and GDNF to the ALS spinal cord.

## Results

### Preclinical studies demonstrate product safety and efficacy

To assess the optimum dose, clinical grade CNS10-NPC-GDNF was injected into the SOD1 rat lumbar spinal cord in a 2-µl volume at five sites at doses of 10,000, 50,000, 100,000 and 250,000 cells in 1 µl as the maximum feasible dose (MFD) that would not clog the delivery cannula. Controls included SOD1 rats receiving vehicle and wild-type rats receiving vehicle or MFD. Animals were euthanized at disease onset, defined by two consecutive Basso Beattie Bresnahan (BBB) motor scores of 17, or at disease endpoint, defined by loss of righting reflex after 30 s.

**Fig. 1:**
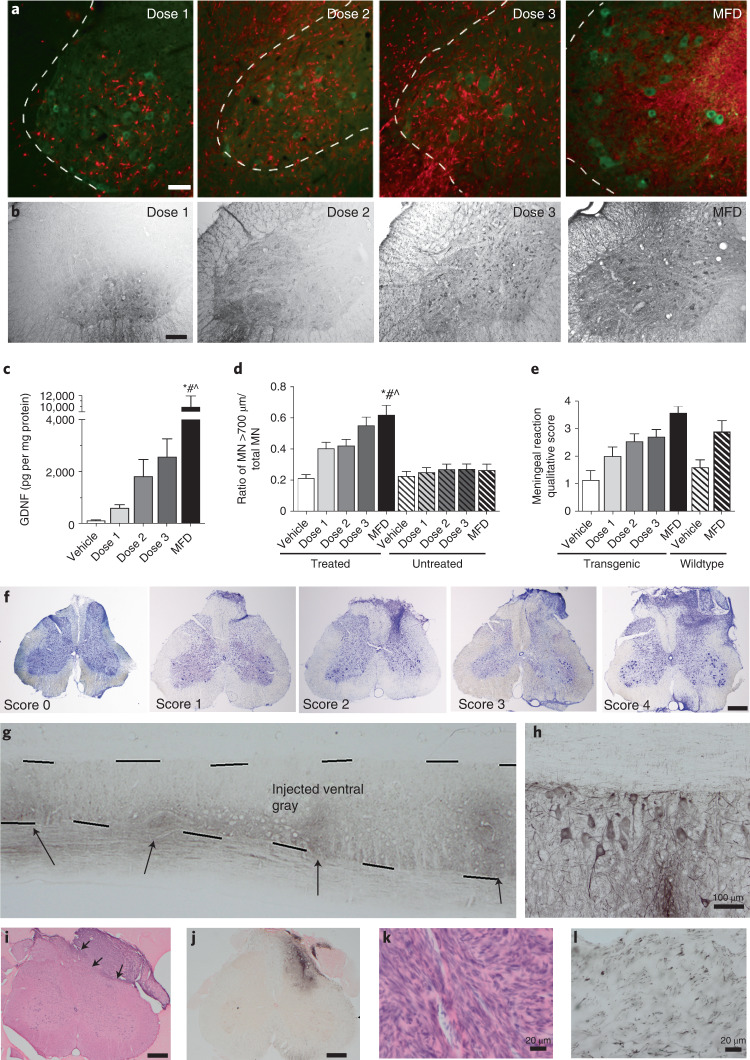
Cell product survives and protects motor neurons and is safe in the spinal cord. **a**, Immunohistochemistry with human-specific nuclear marker SC121 (red) demonstrated dose–response engraftment of CNS10-NPC-GDNF, around host spinal cord ChAT-positive motor neurons (green). **b**,**c**, Human-specific GDNF antibody revealed a large region of staining post-transplantation of all cell doses (**b**), with quantification showing a clear dose-dependence at disease onset (**c**). **d**, Dose-dependent increase in motor neuron (MN) survival. **e**, A meningeal reaction occurred at disease onset in SOD1 and wild-type rats receiving all cell doses and vehicle, and was qualitatively larger at higher doses. **f**, Nissl stain of meningeal reaction at the surgical site in the region of the dorsal root entry, with qualitative score (0–4). Sample size *n* = 15 biologically independent animals for each dose and *n* = 10 for vehicle. **g**,**h**, Immunohistochemistry with human-specific GDNF antibody showed immunodeficient rats had GDNF production at multiple transplant sites for up to 180 d (**g**), with GDNF uptake by host motor neurons (**h**). **i**, H&E stain showed a meningeal reaction associated with the DREZ near the cannula insertion site. **j**–**l**, Immunohistochemistry showed that these structures occasionally had (**j**) the cellular product within them based on a human-specific marker for nestin, (**k**) were positive for Schwann-like cells based on S100b and (**l**) contained numerous small-diameter axons based on a neurofilament heavy stain. Sample size *n* = 12 and *n* = 40 biologically independent animals for 30- and 180-d timepoints, respectively. Scale bars, 75 µm (**a**); 500 µm (**b**,**f**,**i**,**j**); 100 µm (**h**); 20 µm (**k**,**l**). A general linear regression model was used for ELISA (**c**) and was Tukey-adjusted for multiple comparisons. ^*^MFD versus vehicle and MFD versus dose 1, *P* < 0.0001; ^#^MFD versus dose 2, *P* = 0.0027; ^^^MFD versus dose 3 *P* = 0.0009. A mixed-model regression with compound symmetry covariance structure was used for the motor neuron counts (**d**) and Tukey-adjusted for multiple comparisons. ^*^MFD versus vehicle and MFD versus untreated conditions, *P* < 0.0001; ^#^MFD versus dose 1 *P* = 0.01387; ^^^MFD versus dose 2, *P* = 0.02299. Differences were considered significant at the two-sided level of *P* < 0.05. Error bars, ±s.e.m.

**Extended Data Fig. 1:**
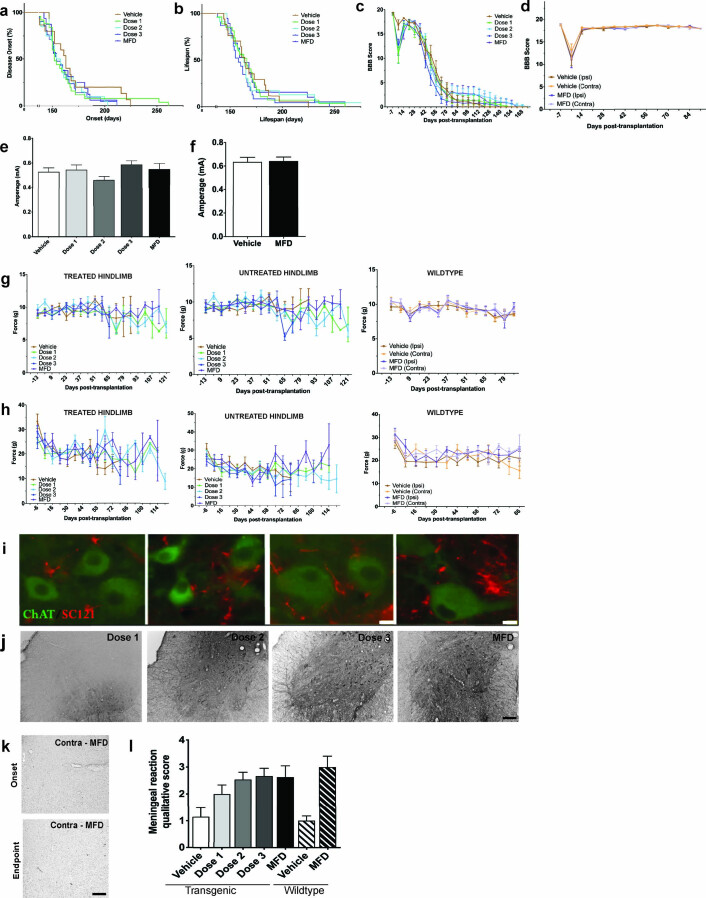
Cell product survival and safety in rodent spinal cord. Cell product survival and safety in rodent spinal cord. Kaplan-Meier curves show no significant differences in (**a**) disease onset or (**b**) lifespan between SOD1 cohorts receiving vehicle or CNS10-NPC-GDNF at Dose 1, Dose 2, Dose 3, or maximal feasible dose (MFD). The BBB score showed (**c**) no difference on functional decline for ipsilateral (treated) hindlimbs over time prior to and following intraspinal injection of vehicle or increasing doses of CNS10-NPC-GDNF and (**d**) no difference on functional decline for ipsilateral and contralateral (untreated) hindlimbs. All groups showed dip at 7-days post-transplantation, considered an artifact of surgical procedure. No cell dose had an effect on ipsilateral (treated) hindlimb pain in SOD1 rats, or MFD in wildtype rats, as demonstrated by the (**e**, **f**) Flinch Jump, (**g**) Randall-Sellito and (**h**) Von Frey tests. (**i**) Immunohistochemistry with human-specific cytoplasmic marker SC121 (red) demonstrated engraftment of CNS10-NPC-GDNF around ChAT-positive motor neurons (green). A human-specific GDNF antibody revealed a large region of staining (**j**) on the transplant side at disease endpoint, but (**k**) not on the contralateral side with MFD at disease onset or endpoint. (**l**) Meningeal reaction occurred in SOD1 and wildtype rats at disease endpoint, receiving all cell doses and receiving vehicle. Sample size n = 15 biologically independent animals for each dose and n = 10 for vehicle. Error bars ± s.e.m. Scale bars: (i) 5 µm; (j, k) 500 µm.

**Extended Data Fig. 2:**
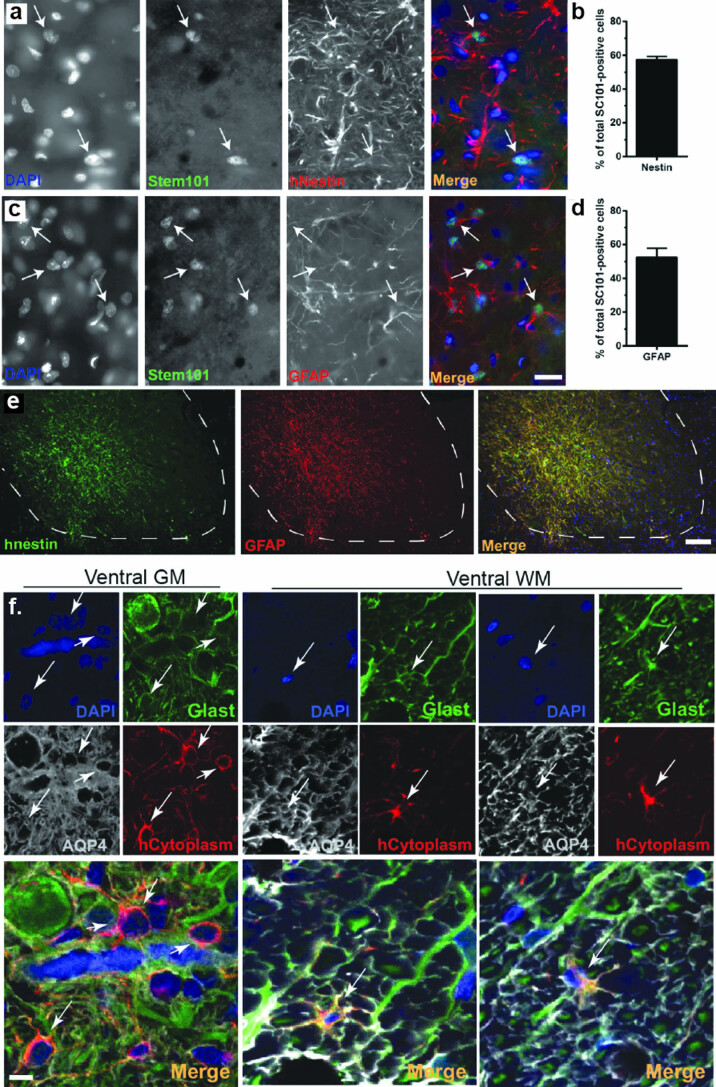
Cell product differentiation into astrocytes. Cell product differentiation into astrocytes. Representative image and quantification of double immunofluorescence for (**a**, **b**) CNS10-NPC-GDNF labeled with Stem101 (nuclear specific, green) and human nestin, a neural progenitor marker, and with (**c**, **d**) GFAP, an astrocyte marker (red). (**e**) Double immunofluorescence shows that many cells are positive for human nestin and GFAP. (**f**) Confocal microscopy images show that CNS10-NPC-GDNF labeled with Stem 121 (cytoplasmic specific, red) co-localized with mature astrocyte markers aquaporin 4 (AQP4) and Glast (green). Nuclei counterstained with DAPI (blue). White arrows indicate CNS10-NPC-GDNF co-expressing the markers. Sample size n = 15 biologically independent animals for each dose and n = 10 for vehicle. Error bars ± s.e.m. Scale bars: (a, c) 10 µm; (e) = 75 µm; (f low mag) 10 µm; (f high mag) 5 µm.

No cell dose had an effect on disease onset or overall lifespan (Extended Data Fig. 1a,b), or on functional decline of the treated hindlimb based on a modified BBB test (Extended Data Fig. 1c), as we have previously shown^25^. Wild-type rats receiving MFD showed no difference in ipsilateral or contralateral hindlimb function compared with rats receiving vehicle (Extended Data Fig. 1d). No cell dose in SOD1 rats, or MFD in wild-type rats, affected ipsilateral or contralateral hindlimb pain, as demonstrated by flinch–jump (Extended Data Fig. 1e,f), Randall–Sellito (Extended Data Fig. 1g) and Von Frey (Extended Data Fig. 1h) tests. The human-specific cytoplasmic marker SC121 showed surviving cells, which surrounded choline acetyl transferase (ChAT)-positive host motor neurons (Fig. 1a and Extended Data Fig. 1i), and a human-specific antibody revealed a large region of GDNF that correlated with cell distribution at disease onset and endpoint (Fig. 1b and Extended Data Fig. 1j), with a clear dose-dependence (Fig. 1c). GDNF was not visible in the untreated hemisphere of animals receiving MFD at onset or endpoint (Extended Data Fig. 1k). Critically, there was a significant dose-dependent increase in motor neuron survival compared with vehicle at disease onset, even at the lowest dose (Fig. 1d), corroborating our previous work^25^. Most CNS10-NPC-GDNF cells remained as nestin-positive neural progenitors or differentiated into glial fibrillary acidic protein (GFAP)-positive astrocytes, with many positive for both markers (Extended Data Fig. 2a–f); no observed cells differentiated into neurons. No animals showed CNS10-NPC-GDNF outside the spinal cord and sciatic nerve based on an analysis of rat tissues using a quantitative polymerase chain reaction (PCR) of human ALU-Y repeats. A meningeal reaction occurred at the surgical site in the region of the DREZ in SOD1 and wild-type rats receiving all cell doses and vehicle, which was qualitatively larger at higher compared with lower doses and compared with vehicle (Fig. 1e,f and Extended Data Fig. 1l).

Next, a tumorigenicity and toxicology Good Laboratory Practice (GLP) study was performed. Immunodeficient rats received unilateral lumbar spinal cord injections of vehicle or CNS10-NPC-GDNF in a 2-µl volume at five sites at a dose of 50,000 or 200,000 cells in 1 µl. Surviving cells at all transplant sites in all animals showed GDNF production for up to 180 d (Fig. 1g), with GDNF uptake by host motor neurons (Fig. 1h). Meningeal reactions again occurred at the DREZ near the cannula insertion site (Fig. 1i), and occasionally contained cellular product based on a human-specific nestin stain (Fig. 1j). S100b-positive Schwann-like cells appeared within the masses (Fig. 1k), possibly induced to proliferate in response to GDNF and/or sprouting of sensory and sympathetic axons in the area based on numerous neurofilament heavy-stained small-diameter axons (Fig. 1l)^36–38^.

**Fig. 2:**
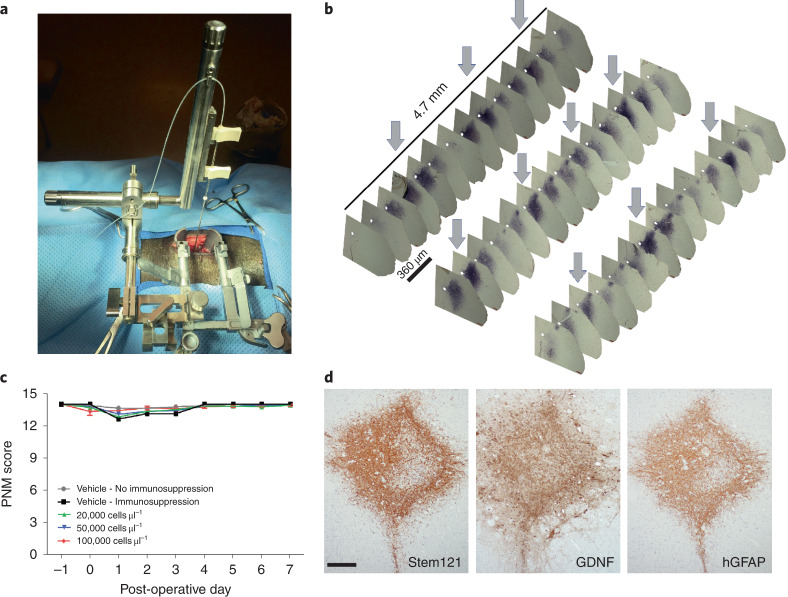
Cell product survives in large animal spinal cord. **a**, Newly developed stereotaxic frame mounted on the Medtronic MAST Quadrant minimally invasive retractor system. **b**, Immunohistochemistry with human-specific GDNF antibody showed robust and widespread GDNF production along the lumbar spinal cord, with arrows highlighting the 2-mm injection intervals. **c**, A GLP study showed that only a few animals exhibited lower PNM scores during the first 3 d post-surgery, with all animals presenting PNM scores of at least 13 from day 4 until designated termination. Sample size *n* = 52 biologically independent animals. **d**, Immunohistochemistry with human-specific antibodies demonstrated cell survival (Stem121), GDNF production and differentiation into GFAP-positive astrocytes. Scale bar, 200 µm.

To deliver CNS10-NPC-GDNF to the patient spinal cord, a new stereotaxic frame mounted on the Medtronic MAST Quadrant minimally invasive retractor system was developed and initially evaluated in a large animal mini-pig model (Fig. 2a). CNS10-NPC-GDNF injected in a 10-µl volume to ten sites at a dose of 20,000, 50,000 and 200,000 cells in 1 µl survived and a human-specific antibody showed widespread GDNF production in the ventral horn of the lumbar spinal cord (Fig. 2b). A safety GLP study was performed with mini-pigs receiving vehicle or CNS10-NPC-GDNF in a 10-µl volume to ten sites at a dose of 20,000, 50,000 and 100,000 cells in 1 µl. A 14-point scale porcine neurological motor (PNM) score showed that only a few animals exhibited reduced motor function during the first 3 d post-surgery, with all animals presenting scores of 13 or 14 from day 4 until termination day (Fig. 2c). Human-specific antibodies demonstrated cell survival, neural progenitor cells, astrocytes and GDNF production in the ventral horn (Fig. 2d). No neuromas were observed, even with the highest cell dose and up to 31 d post-injection. Collectively, these preclinical studies allowed the filing of an IND to the FDA, that approved product translation to a phase 1/2a clinical trial.

### Phase 1/2a trial enrollment and cell delivery

Twenty-one patients were selected for screening and 18 were randomized to this trial after eligibility was met, including diagnosis of ALS based on El Escorial Criteria for lab-supported probable, probable or definite ALS, with disease duration ≤3 years, evidence of lumbar spinal motor neuron involvement either clinically or on electromyography, and a supine forced vital capacity of >60% (Table 1). Clinical evidence included findings of muscle atrophy, accompanied by fasciculations and weakness indicative of lower motor neuron (LMN) involvement. All enrolled participants had an electromyography that confirmed clinical findings were accompanied by denervation. The first dose cohort (*n* = 9) received ten unilateral injections comprising of 200,000 cells per site for a total of 2,000,000 cells and the second dose cohort (*n* = 9) received 500,000 cells per site for a total of 5,000,000 cells. Targeting was based on magnetic resonance imaging (MRI) of the entire cord with the first injection being ~6.2 cm from the conus, 1–2 mm medial from the DREZ and each subsequent injection being 2 mm apart moving caudally. Depth of targeting was based on MRI and the use of one of three cannula lengths (3.8, 4.3, 4.7 mm). To avoid damage to the remaining motor neurons, cells were not injected directly into the ventral horn, but instead targeted the transition zone between the dorsal and ventral horns, with our preclinical rodent data predicting that cells would migrate ventrally into the motor neuron pool. Surgeries and product delivery were performed without adverse events. The hospital stay was 5 d and participants were followed for one year with assessments at months 1, 2, 3, 6, 9 and 12 post-transplantation. All participants were followed for long-term vitality status.

**Table 1:** Details of the 18 enrolled trial participants

Parameter	All participants (*n* = 18)	High dose (*n* = 9)	Low dose (*n* = 9)
Age (years)	57.5 ± 10.5	56.5 ± 11.4	58.4 ± 10.1
Sex			
Female	10 (55.6%)	5 (55.6%)	5 (55.6%)
Male	8 (44.4%)	4 (44.4%)	4 (44.4%)
Ethnicity			
Hispanic or Latino	2 (11.1%)	2 (22.2%)	0 (0%)
Non-Hispanic or Latino	16 (88.9%)	7 (77.8%)	9 (100%)
Race			
White	18 (100%)	9 (100%)	9 (100%)
Forced vital capacity (max %-pred)	92.1 ± 22.0	95.1 ± 22.8	89.1 ± 22.2
Weight (kg)	83.5 ± 13.8	85.4 ± 17.4	81.5 ± 9.78
Months since symptom onset	18.8 ± 8.1	16.7 ± 5.0	20.8 ± 10.2
Months since diagnosis	10.7 ± 5.8	11.7 ± 5.3	9.6 ± 6.5
BMI (kg m^−^^2^)	27.7 ± 3.47	28.2 ± 4.00	27.3 ± 3.01
Onset site			
Bulbar	2 (11.1%)	1 (11.1%)	1 (11.1%)
Limb	16 (88.9%)	8 (88.9%)	8 (88.9%)
Taking riluzole			
No	6 (33.3%)	5 (55.6%)	1 (11.1%)
Yes	12 (66.7%)	4 (44.4%)	8 (88.9%)
Family history of ALS			
No	15 (83.3%)	7 (77.8%)	8 (88.9%)
Unknown	2 (11.1%)	2 (22.2%)	0 (0%)
Yes	1 (5.6%)	0 (0.0%)	1 (11.1%)
Lumbar UMN clinical signs			
No	3 (16.7%)	2 (22.2%)	1 (11.1%)
Yes	15 (83.3%)	7 (77.8%)	8 (88.9%)
Lumbar LMN clinical signs^a^			
No	5 (27.8%)	1 (11.1%)	4 (44.4%)
Yes	13 (72.2%)	8 (88.9%)	5 (55.6%)
Lumbar electromyography signs	18 (100%)	9 (100%)	9 (100%)
ALSFRS-R total score	36.8 ± 4.7	37.9 ± 5.3	35.8 ± 4.0
Bulbar subscore	10.6 ± 2.23	10.9 ± 1.96	10.2 ± 2.54
Fine motor subscore	8.83 ± 2.36	9.78 ± 2.33	7.89 ± 2.09
Gross motor subscore	6.67 ± 2.00	6.44 ± 1.88	6.89 ± 2.20
Respiratory subscore	10.8 ± 1.70	10.8 ± 1.72	10.8 ± 1.79
Post-operative month at death	23.9 ± 8.7	25.9 ± 8.3	22.3 ± 9.3

Mean ± s.d.

^a^Examples of lumbar LMN clinical signs include weakness, flaccidity, atrophy and fasciculations.

### Trial meets primary outcome measure of safety

In the immediate post-operative period, 89% of the low-dose cohort and 67% of the high-dose cohort reported dysesthesia and/or paresthesia and/or pain/discomfort in the region innervated by the surgery site, approximately the L2–L4 dermatome corresponding to the anterior thigh, which was likely due to needle passes during injections. In some cases, the neuropathic pain subsided by hospital discharge, and the remainder of participants were managed with primarily gabapentin (Supplementary Tables 1 and 2) or prescription analgesics (participants 101, 103, 113, 114). In nine participants, lower extremity pain on the treated side lasted more than 6 months, with five participants reporting a severity of ≥5. For this long-term pain, five participants were treated with gabapentin, although rarely at the level used for severe neuropathic pain, and/or with other prescription analgesics, with four participants requiring no use of prescription pain medication.

Because CNS10-NPC-GDNF is an allogenic product, all participants received immunosuppression, of whom 12 had no changes or disruptions over the 1-year course, whereas 6 had an altered course (Table 2). Participants 105 and 106 discontinued immunosuppression after months 4 and 9, respectively, because of adverse events; participant 118 voluntarily stopped for ~1 month; participants 109 and 102 had minor disruptions in immunosuppression due to adverse events but completed the 1-year course; participant 101 developed headaches and substituted tacrolimus with cyclosporine at month 2, until month 12.

**Table 2:** Details of the 18 trial participants following treatment

Dose	Participant	DSA	Immunosuppression		RCL	Anti-GDNF Ab	GDNF in CSF/serum	Post-operative month at death
			Duration (months)	Modifications				
Low dose	101	N	12	Major	N	N	BLOQ	42
	102	N	12	Minor	N	N	BLOQ	20
	103	N	12	None	N	N	BLOQ	28
	104	N	12	None	N	N	BLOQ	15
	105	N	4	Major	N	N	BLOQ	24
	106	N	9	Major	N	N	BLOQ	14
	107	N	12	None	N	N	BLOQ	15
	108	N	12	None	N	N	BLOQ	
	109	N	12	Minor	N	N	BLOQ	21
High dose	110	N	12	None	N	N	BLOQ	
	111	N	12	None	N	N	BLOQ	18
	112	N	12	None	N	N	BLOQ	32
	113	N	12	None	N	N	BLOQ	38
	114	P	12	None	N	N	BLOQ	14
	115	N	12	None	N	N	BLOQ	26
	116	N	12	None	N	N	BLOQ	
	117	N	12	None	N	N	BLOQ	
	118	N	12	Major	N	N	BLOQ	29

Ab, antibodies; BLOQ, below limits of quantification; CSF, cerebral spinal fluid; N, negative; P, positive; RCL, replication-competent lentivirus.

**Fig. 3:**
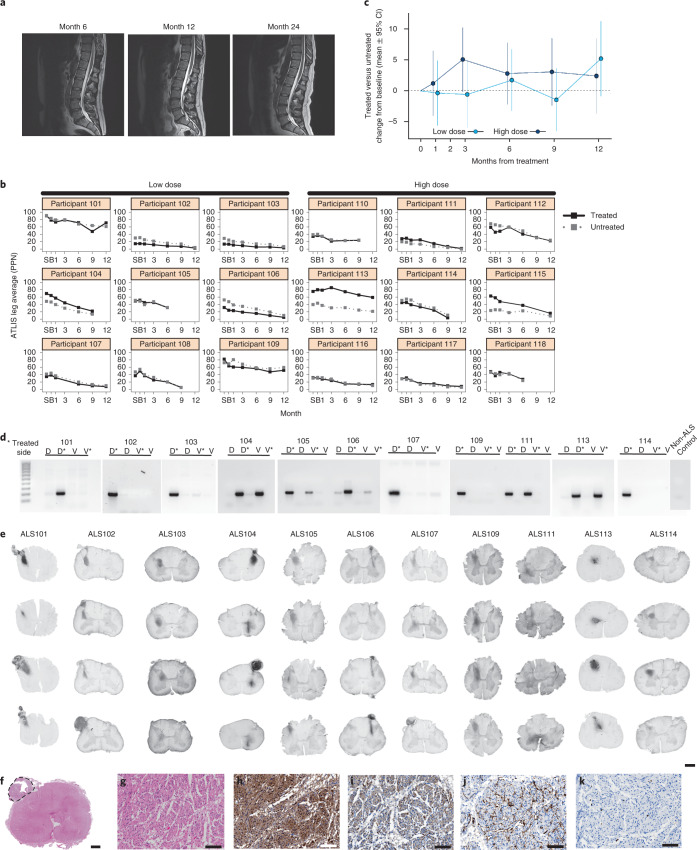
Clinical trial meets safety endpoint and shows cell product survival and GDNF production. **a**, MRI scans were normal throughout the study for all participants, apart from one with mild increased T2 hyperintensity in the area of the graft, that resolved after surgery. **b**, Measuring the decline in strength for the quadriceps (knee extension) over time for all participants from both dose cohorts showed that both legs became weaker, at varying rates. **c**, Although not statistically significant, the largest relative difference in the treated leg compared with the untreated leg in the low-dose cohort occurred at 12 months. The high-dose cohort showed a consistently slower rate of decline in the treated leg at all time points. **d**, Cell survival was shown in all participants by nested PCR, performed on four quadrants of the spinal cord for dorsal (D) and ventral (V) on both the treated (asterisk) and untreated sides. **e**, Immunohistochemistry revealed GDNF production in all participants. **f**,**g**, H&E staining demonstrated an (**f**) exophytic mass composed of (**g**) spindle cells arranged in a haphazard and intersecting fascicular growth pattern. **h**, Immunohistochemistry showed that the spindle cells were positive for S100, consistent with a Schwann cell origin. **i**, Neurofilament staining showed numerous punctae and irregularly shaped structures consistent with disorganized and regenerative axons mixed with the Schwann cells. **j**,**k**, CD34 staining showed (**j**) normal-appearing blood vessels within the mass and (**k**) cells were only infrequently Ki67-positive. Scale bar, 1 mm (**e**, **f**); 100 µm (**g**–**k**). Source data

**Extended Data Fig. 3:**
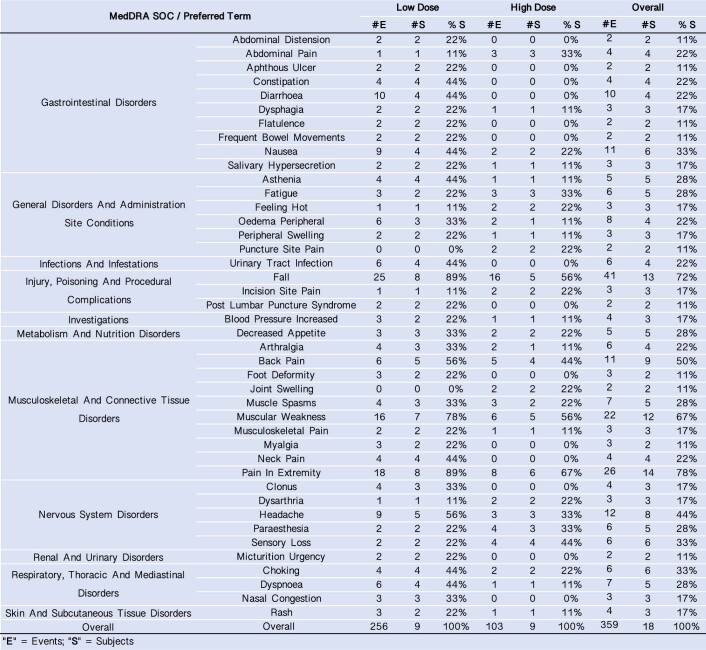
Adverse events. Adverse events reported by at least 20% of participants. #e = number of events; #s = number of participants.

**Extended Data Fig. 4:**
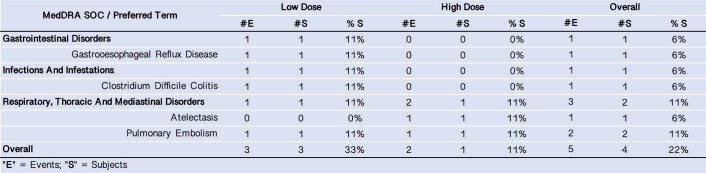
Serious adverse events. No serious adverse events during the trial were attributed to the product.

Common adverse events included falls, extremity pain, nausea, back pain and muscular weakness typically associated with ALS, immunosuppression and surgery. All adverse events reported by at least 20% of participants are provided in Extended Data Fig. 3. MRI of the lumbosacral enlargement in the graft area immediately post-surgery showed mild increased T2 hyperintensity in only one participant (102), which resolved by 48 h after surgery; otherwise all MRI scans were normal throughout the study (up to 24 months), with the laminectomy delineating the graft region (Fig. 3a). Donor-specific antibodies (DSA) were detected only in participant 114 (Table 2) and were positive at baseline (DR17 and DR18 (> 2,500–3,750 mean fluorescence intensity (MFI), Class II panel reactive antibody (CII PRA 45%) and increased in specificity throughout the study (DR17 >7,500–8,750 MFI, DR18 >6,250–7,500 MFI, CII PRA 79%) including a de novo DSA (DRB3:01 >5,000–6,250 MFI). No participant had detectable replication-competent lentivirus or GDNF in the cerebral spinal fluid (Table 2). No serious adverse events during the trial were attributed to the product (Extended Data Fig. 4). The trial sample size was selected to ensure >80% power to detect adverse events or other safety outcomes that had an expected incidence of at least 9%. These results show that this phase 1/2a trial reached its primary endpoint of safety.

### Secondary outcome measures

**Extended Data Fig. 5:**
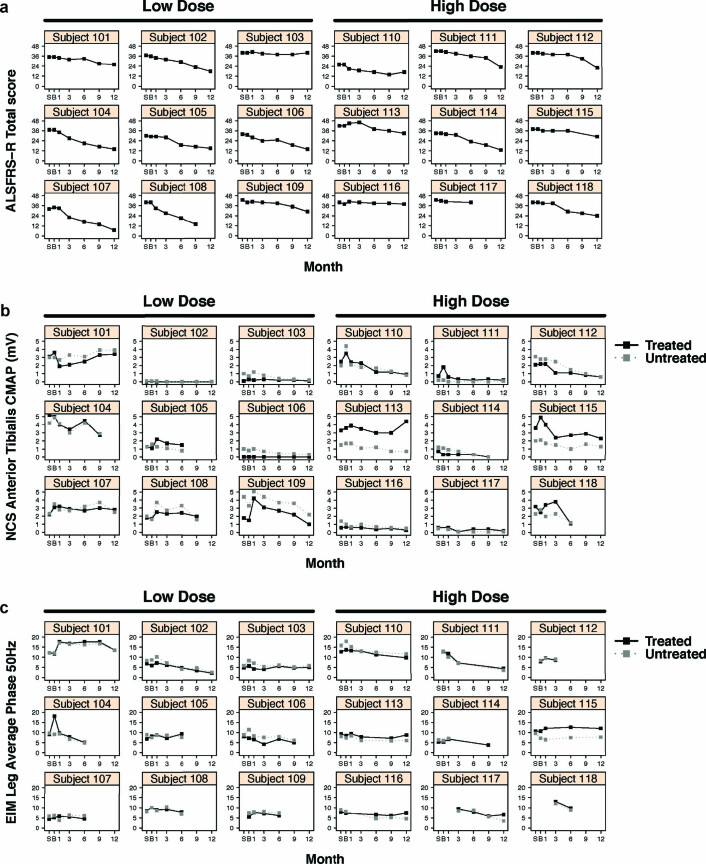
Additional measures collected for all participants. Additional measures collected over the course of the study for all participants included (**a**) ALSFRS-R score, (**b**) compound motor action potential (CMAP) of the tibialis anterior and (**c**) electrical impedance myography (EIM).

**Extended Data Fig. 6:**
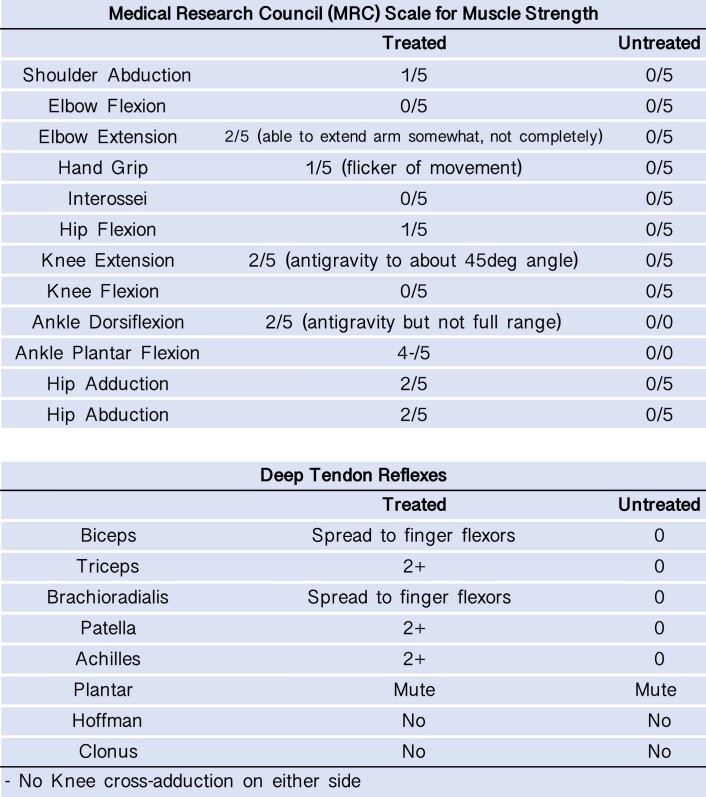
Additional tests collected for Participant 113 at 3 years post-transplantation. Muscle strength, based on Medical Research Council (MRC) scale, and deep tendon reflexes were collected as additional tests only for Participant 113, after trial completion at 3 years post-transplantation, as ATLIS was not available at this timepoint.

The ALS Functional Rating Scale Revised (ALSFRS-R) score is a patient-reported, clinician-assessed, 48-point scale measuring overall functional status. The total score had a variable range at screening (mean 36, range 29–40), and participants progressed at a mean rate of 1.2 points per month (95% confidence interval (Cl) −1.5 to −0.9 points per month), typical of ALS trial participants (Extended Data Fig. 5a). The unilateral trial design allowed direct comparison of treated and untreated leg muscle strength, removing the variability in bilateral studies due to patient variability in strength decline. We have shown that muscle groups on each side of the body have remarkably similar progression rates in individual patients with ALS^39^. Therefore, we predicted that identical progression rates would indicate no treatment effect, whereas a slower decline in the treated leg compared with the untreated leg would suggest a positive graft effect. Fixed dynamometry using an accurate test of limb isometric strength (ATLIS) device measured strength over time in the treated and untreated leg for three muscle groups, knee extensors (L2–L4 myotomes), ankle dorsiflexors (L4–L5 myotome) and knee flexors (S1 myotome). Percent of predicted normal strength of each muscle was averaged for a total leg score, which decreased in all participants because of disease progression (Fig. 3b). On average, treated legs lost strength at a numerically slower rate than untreated legs (adjusted mean difference across cohorts = 0.22 percent-normal per month, 95% CI −0.09 to 0.53, *P* = 0.16). Although no differences were statistically significant, the largest relative difference in the treated leg compared with the untreated leg in the low-dose cohort occurred at 12 months and the high-dose cohort showed a consistently slower rate of decline in the treated leg at all time points (Fig. 3c). Participants were enrolled into an optional follow-on protocol for extended monitoring. One participant (113) showed markedly preserved function on the treated side at 3 years post-transplantation (Supplementary Video 1 and Extended Data Fig. 6). This participant also had some asymmetrically preserved hand function on the same side that was unlikely to be associated with transplanted cells, although the extent of function preservation in the hand was less than in the leg (Extended Data Fig. 6). Another set of functional testing was used to compare innervation of the treated and untreated leg in all participants, using compound motor action potential (CMAP) of the tibialis anterior and electrical impedance myography (EIM), with most outcome measures showing high variability over time and none showing any significant positive or negative changes in response to treatment (Extended Data Fig. 5b,c).

### Post-mortem studies

**Extended Data Fig. 7:**
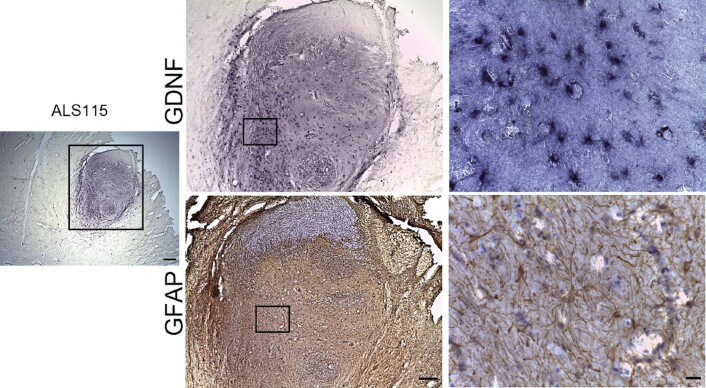
Astrocytes and GDNF within the transplants. Immunohistochemistry shows GFAP-positive astrocytes and GDNF-positive cells within solid oval transplants. Scale bars: Panels left 400 μm; middle 200 μm; right 20 μm.

Eight participants from the low-dose cohort and six from the high-dose cohort died of ALS disease progression between 14 and 42 months after cell transplantation (Table 2). The brains and spinal cords were collected from 13 participants within 24 h, and analyzed for graft survival and pathological changes. Nested PCR against the murine phosphoglycerate kinase (mPGK) promoter and human GDNF sequences contained within the GDNF lentivirus construct was performed in the dorsal and ventral spinal cord on the treated and untreated sides. A DNA signal confirmed cell survival, which was only detected on the transplanted side (denoted by the asterisk) and more prominently in the dorsal segment (Fig. 3d). A control ALS patient spinal cord not receiving a transplant had no detectable DNA signal. Immunohistochemistry revealed GDNF product in the spinal cord of all 13 participants, only on the transplanted side (Fig. 3e). Participants 104 and 111 showed robust GDNF staining in the ventral horn; however, most participants showed GDNF staining primarily in the dorsal or intermediate zone of the spinal cord. Occasionally solid oval transplants with clearly demarcated borders were observed containing GDNF-positive cells and GFAP-positive astrocytes (Extended Data Fig. 7).

**Extended Data Fig. 8:**
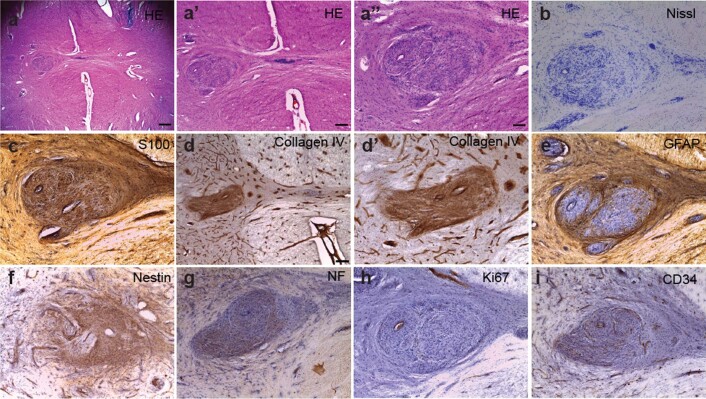
Neuromas within the Virchow-Robin space. In some cases, neuromas extended down into the Virchow-Robin space of the dorsal and ventral horns of the spinal cord, shown with (**a** – **a”**) H&E and (**b**) Nissl. Structures stained positive for (**c**) S100 and (**d**) Collagen IV, and (**e**) negative for GFAP. Structures stained positive for (**f**) nestin and (**g**) neurofilament (NF). (**h**) There was no observed staining for Ki67. (**i**) CD34 demonstrated normal appearing blood vessels within the structures. Scale bars: (a) 400 μm; (a’, d) 200 μm; (a”) 100 μm; (b, c, d’, e-i) are all the same 100 μm scale bar size shown in a”.

Nine participants had rounded structures around the DREZs at some injection sites (Fig. 3f), some which showed GDNF production, for example participants 102 and 104 (Fig. 3e). Detailed investigation by a neuropathologist of all cases stained with hematoxylin and eosin (H&E) demonstrated an exophytic mass composed of spindle cells arranged in a haphazard and intersecting fascicular growth pattern (Fig. 3g). Immunohistochemistry showed that the spindle cells were strongly and diffusely positive for S100b (Fig. 3h) consistent with abundant Schwann cells, which was mixed with neurofilament staining of numerous punctae and irregularly shaped structures consistent with disorganized and regenerative axons (Fig. 3i). CD34 highlighted normal-appearing blood vessels within the mass (Fig. 3j) and cells were only infrequently Ki67-positive (Fig. 3k), indicating a very low cell proliferation rate. Nested PCR against the mPGK promoter showed CNS10-NPC-GDNF in some, but not all cases. These were interpreted to be neuromas at the injection site, and were not detected on the MRI scans (with or without gadolinium contrast) for any participant despite their appearance on histology. In four participants to date, neuromas extended into the Virchow–Robin space of the spinal cord, which was prominent in the ventral horn of participant 113 (Extended Data Fig. 8a,b). These structures were positive for S100b and Collagen IV but GFAP-negative (Extended Data Fig. 8c–e), consistent with the Schwann cell and non-CNS origin. Intraparenchymal neuromas stained for nestin and, similar to exophytic masses described above, neurofilament staining was consistent with disorganized and regenerative axons mixed with Schwann cells (Extended Data Fig. 8f,g). CD34 demonstrated normal-appearing blood vessels within the structures and Ki67-positive cells were absent or infrequent (Extended Data Fig. 8h,i).

**Fig. 4:**
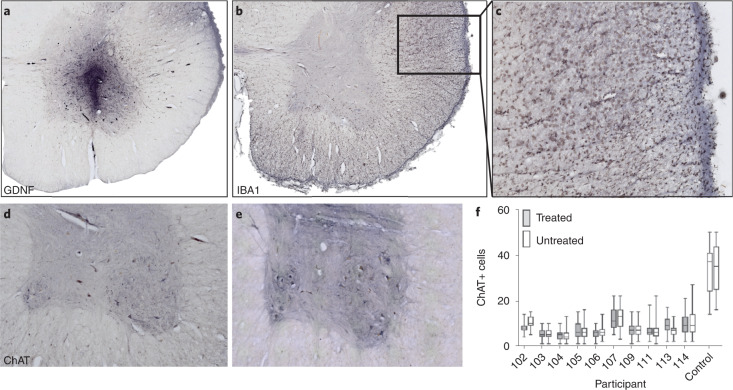
Motor neurons were similar between treated and untreated sides. **a**,**b**, Immunohistochemistry showed graft survival with a high level of GDNF (**a**), yet very low levels of IBA1 staining (**b**). **c**, Descending motor neuron tracts showed an inflammatory response with hypertrophic microglia and IBA1 staining. **d**,**e**, Immunohistochemistry showed that ChAT-positive motor neurons were (**d**) lost in the ALS lumbar spinal cord and (**e**) preserved in the control spinal cord. **f**, The treated and untreated spinal cord sides showed similar numbers of ChAT-positive motor neurons across participants; motor neurons were significantly lower in participant 102 (*p* = 0.0001) and significantly higher (*p* = 0.0013) in 113 on the treated compared to untreated side. Box plots depict median and 25th to 75th percentiles, with min and max whiskers. Sample size *n* = 47 (on average) independent participant spinal cord sections, *n* = 25 independent control spinal cord sections. Magnification, ×2.5 (**a**,**b**); ×20 (**c**); ×5 (**d**,**e**).

**Extended Data Fig. 9:**
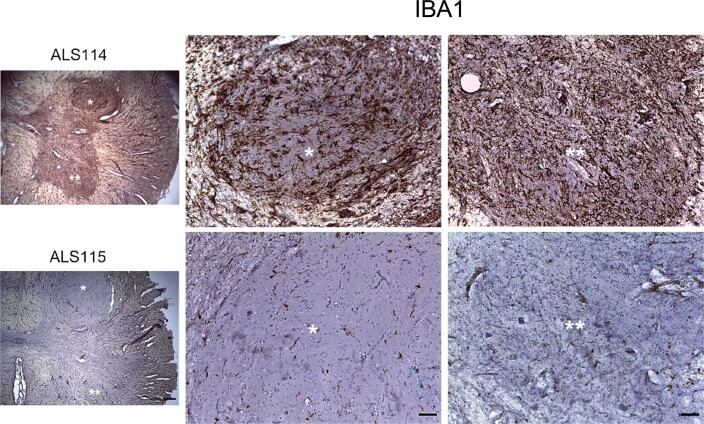
IBA1 staining in Participant 114. Immunohistochemistry shows IBA1 staining in Participant 114, which is not observed in other participants, with a representative image from participant 115. Scale bars: Panels left 400 μm; middle and right 100 μm.

All participants were off protocol and immunosuppression for 2–30 months until death (Table 2). Immunohistochemistry with the microglia marker, IBA1, demonstrated little or no local immune reaction in the spinal cord, even in regions of graft survival and intense GDNF staining (Fig. 4a,b). By contrast to the lack of inflammatory markers around the transplant area, regions containing descending motor neuron tracts showed an extensive microglial response with hypertrophic microglia and robust IBA1 staining, likely in response to upper motor neuron (UMN) death and lateral column degeneration in the ALS spinal cord (Fig. 4c). The one exception was participant 114, who showed massive IBA1 activity throughout the spinal cord gray and white matter, suggesting a widespread reaction to transplanted cells compared with other participants (Extended Data Fig. 9). Interestingly, 114 was the only participant having donor-specific antibodies, likely contributing to reactive levels.

Immunohistochemistry showed, as expected, that ChAT-positive spinal cord motor neurons were dramatically reduced in ALS participants compared with a non-ALS control, containing many large motor neurons with an average of 35 motor neurons on one side of a single section (Fig. 4d–f). Motor neuron numbers on the treated side were significantly lower in participant 102 and significantly higher in participant 113; however, treated and untreated sides showed no overall significant difference across participants (Fig. 4f).

## Discussion

This is the first study to show that allogeneic neural progenitors engineered to release GDNF can be safely transplanted into the human CNS. Remarkably, graft survival and GDNF production were evident in all 13 participants for up to 42 months after transplantation, even though immune suppression was for 1 year, with no signs of rejection or an inflammatory reaction in all but one case. Neural progenitor grafts in animal models and in ALS patients showed differentiation into astrocytes. The primary endpoint of safety was reached, with no serious adverse events attributed to the product. This is proof-of-concept that a single delivery of genetically modified neural progenitor cells showed sustained survival and release of a protein product.

Previous trials (NCT01348451; NCT01730716) most similar to this one transplanted fetal human spinal cord-derived neural stem cells (NSI-566) into the ALS lumbar and cervical spinal cords^9–11^. Unlike CNS10-NPC-GDNF cells, which produce mainly astrocytes following transplantation, NSI-566 cells differentiated mainly into interneurons and were not engineered to produce GDNF. Although there was some cell survival, there was no delay in disease progression; however, these studies did not use a unilateral design and were not powered for efficacy.

ALS is a heterogeneous disease in which progression rates in individual patients can vary extensively^40^. However, we have shown in a large patient cohort that the progression rate in individual patients with ALS is remarkably similar for functional muscle groups on each side of the body^39^. Directly comparing treated and untreated legs for individual patients removes the variability seen in bilateral studies due to different progression rates. The current unilateral trial had participant and study team blinded to which leg was treated, although post-operative pain may have impacted blinding in some cases. There was no negative effect of the transplant on progression rate of the treated leg in both low- and high-dose cohorts compared with the untreated leg, supporting the safety of the intervention as possible damage associated with cell transplantation did not exacerbate symptoms. Another unique aspect of this trial is the focal cell and protein delivery, targeting specific sites within the CNS, compared with systemic drug delivery, particularly for GDNF, as peripheral delivery has been associated with adverse effects^41^.

To avoid disrupting diseased motor neurons in the ventral horn, we targeted the spinal cord region just above. Graft survival was encouraging and remarkably similar to our preclinical studies in rodents^16,25^ and pigs^22^. However, most grafts were observed in the dorsal horn, with less migration into the ventral horn than predicted from the preclinical rodent studies, and cells likely refluxed up the cannula. This dorsal location perhaps contributed to the chronic neuropathic-type pain^42^ that was reported by 50% of participants and managed by medication. The pain profile observed here will be an important risk versus benefit to communicate in future spinal cord injection studies, which will target more ventral regions to mitigate cell reflux into the dorsal horn.

Although the brain is thought to be immune privileged, most fetal transplant trials for Parkinson’s disease used immunosuppression for at least 6 months, with transplants typically surviving without long-term immunosuppression^43–45^. Our trial protocol was a 12-month immunosuppression course, although some participants stopped earlier. Even though CNS10-NPC-GDNF is allogeneic, expanded in culture and genetically modified, all participants had surviving grafts, suggesting that continual immunosuppression is not required. In one case, although there was graft survival, there were also signs of an inflammatory microglial reaction, similar to findings after detailed analysis of fetal grafts^46^. Interestingly, this was the only participant with DSA at the trial start, which may have mounted a directed immune response. The contribution of DSA to an immune response was not assessed in fetal transplant trials. Future trials with allogeneic neural progenitors should likely include a period of immunosuppression post-grafting and could consider DSA as an exclusion criteria.

Although most transplants were located dorsally, we assessed motor neuron survival in the ventral horn, which showed no overall increase or decrease on the treated versus untreated side. Participant 102 actually showed a significant decline in motor neurons on the treated side, which is likely attributed to the fact that this participant had almost no remaining leg function on the treated side at trial onset and only had a transplant in the dorsal horn. By contrast, participant 113 with both dorsal and ventral horn transplants, showed a significant increase in motor neurons on the treated side, which could have contributed to the remaining function observed in the treated leg. Although preclinical data demonstrated that CNS10-NPC-GDNF protected host motor neurons, more patients with engraftment around motor neurons in the ventral horn are required to promote motor neuron survival in the human ALS spinal cord.

Clinical MRI scans showed no spinal cord abnormalities, yet neuromas ranging from 1 to 3 mm were frequently observed post-mortem at several injection sites within the fiber bundles of the DREZs. Neuromas are benign growths within the dorsal roots composed of dividing Schwann cells in the peripheral nervous system. Host Schwann cells, along with intermixed CNS10-NPC-GDNF in some instances, were likely the source of the GDNF staining in the neuromas^47^. The consistent location of these meningeal reactions may be related to damage at the dorsal roots, as injections were made through a dorsal laminectomy. However, trials with NSI-566 cell delivery did not report neuromas even though a very similar neurosurgical approach was used (J. Glass, personal communication). This strongly suggests the neuromas are due to a combination of damage that induced proliferation of host Schwann cells and sprouting of host small axons in the vicinity of the injection site, which was perhaps augmented by growth factor release from the cellular product^36–38^. Similar meningeal masses occurred in the rodent studies at the DREZ; however, none were observed in large animal studies, which may be due to the short 30-d survival time and potentially less reflux with deeper cell delivery into the ventral horn. In some cases, the neuroma was also seen in the spinal cord within Virchow–Robin spaces that surround the walls of blood vessels^48^. The presence of neuromas did not always associate with pain/discomfort or reduced motor function. Supporting this is participant 113 who had a large neuroma within the Virchow–Robin and yet maintained some leg function on the treated side.

This phase 1/2a trial determined the safety of delivering a cell and gene therapy product to the ALS spinal cord; however, there were some limitations. Although targeting only one leg removes the variability seen in bilateral studies due to different progression rates in patients, this unilateral design is not expected to affect the overall ALSFRS-R score and functional status. However, we believe that even preservation of strength in one limb would be considered impactful by patients and clinicians, and could shape strategies for additional delivery in the future. Although this trial was blinded as to the injection side, study staff made a correct guess about treatment side for 11 participants based on the sensory changes. Among participants, 14 of 18 guessed the treated side: nine based on presence of sensory changes and five based on perceived improvement of the treated leg. Although unilateral pain may have impacted some blinding, all possible measures were followed to ensure that blinding was maintained during the study course. CNS10-NPC-GDNF were delivered to the intermediate zone to avoid directly injecting into the diseased motor neuron pool within the ventral horn. Based on rodent studies showing enhanced cell migration with CNS damage^19,49^, we believed that cells would migrate down to the ventral horn; however, most grafts were within the dorsal spinal cord. Future trials that target the ventral horn and earlier stages of disease could optimize the protective effects on motor neurons and function. Avoiding dorsal horn transplants may also attenuate extended pain, which will be important for optimal blinding in efficacy studies. Finally, deeper targeting in the ventral horn may decrease cell reflux up the needle track and hence reduce the risk of neuroma formation at the DREZ. A critical consideration for future trials and remaining participants in this study will be careful following of any neuroma formation with imaging and post-mortem information when available to further understand the long-term consequences of such structures.

This combined stem cell and gene therapy trial for ALS shows that CNS10-NPC-GDNF can be safely delivered to the lumbar spinal cord and produce GDNF for over 3 years with no negative effects on leg function, thus providing promise for patients with this relentless disease and no effective treatment. Ultimately, treating multiple sites may enhance clinically meaningful effects, and this therapeutic approach is being considered for the cervical spinal cord and motor cortex, two regions showing benefit from these cells in ALS models^28,50^. Indeed, a phase 1/2a clinical trial is now delivering CNS10-NPC-GDNF to the motor cortex of patients with ALS (NCT05306457). In addition, we have a phase 1/2a clinical trial delivering CNS10-NPC to patients with retinitis pigmentosa (NCT04284293)^24^ and are performing IND-enabling studies to deliver CNS10-NPC-GDNF to patients with Parkinson’s disease. Given encouraging outcomes from this initial trial, a combined cell and gene therapy approach holds great promise as a therapeutic option for ALS and other neurodegenerative diseases.

## Methods

### CNS10-NPC-GDNF production

cGMP generation and expansion of CNS10-NPC-GDNF product has been described extensively^35^. A single human fetal cortical sample was collected and expanded in epidermal growth factor and fibroblast growth factor-2 for 14 passages as free-floating aggregates, termed neurospheres, which are passaged using a chopping method^51^. Neurospheres were transferred to a cGMP facility (WCBF, Waisman Center, Madison, WI), expanded for five passages and cryopreserved as the master cell bank (termed CNS10-NPC), which underwent full adventitious agent testing. The master cell bank was expanded, under GMP-compliance (City of Hope, Duarte, CA) and at passage 27, cells were dissociated to a single-cell suspension using enzymatic (TrypLE) treatment and trituration, and transduced with cGMP-grade Lenti-SIN-WP-mPGK-GDNF (Indiana University) at 0.125 pg of p24 per cell. Cells re-formed into neurospheres that were expanded and collected at passage 29, dissociated with TrypLE, resuspended in Cell Freezing medium (dimethylsulfoxide, serum-free 1×) and frozen in a control rate freeze. The final product, CNS10-NPC-GDNF, was banked as the clinical cell lot, with 1,321 vials, containing 5 × 10^6^ cells per vial in a 1-ml volume and stored in the vapor phase of liquid nitrogen. CNS10-NPC-GDNF underwent fluorescence in situ hybridization analysis, chromosome analysis with banding and composite lot release testing.

### Dose ranging

Cell lines and protocols were used in accordance with the guidelines approved by the stem cell research oversight committee (SCRO) and institutional review board (IRB) under the auspice IRB-SCRO Protocols 21505 and 29996. Rat work was performed following the guidelines of the Cedars-Sinai Medical Center Institutional Animal Care and Use Committee (IACUC) under protocol number 4260. Male and female transgenic rats (NTac:SDTg (SOD1^G93A^) L26H) and wild-type littermates (Taconics) were maintained as a colony by in-house breeding with Sprague–Dawley females (Taconics), and genotyped with Takara ex taq (Clonetech). Transgenics were assigned to a treatment cohort: 10,000 cells  (dose 1, *n* = 15), 50,000 cells  (dose 2, *n* = 15), 100,000 cells  (dose 3, *n* = 15) and 250,000 cells in a volume of 1 µl (MFD, *n* = 15) or vehicle (*n* = 10). Wild-type animals were assigned to vehicle and MFD groups, and euthanasia was aged-matched to transgenics. Numbers allowed for two or three animals to be lost per group, with statistical justification based on previous publications^17,26^. A subset of animals euthanized before study completion were excluded. If disease occurred because of forelimb paralysis, the animal was transferred from the endpoint to the onset group. Euthanasia took place at disease onset (defined by consecutive BBB score of ≤17 on either hindlimb) or endpoint (defined by loss of righting reflex on either side, within 30 s). For protein analysis, tissue was collected and fast frozen in liquid nitrogen. For immunohistochemical analysis, animals were perfused with 4% paraformaldehyde and 4% sucrose in 0.1 M phosphate buffer.

### GLP toxicology and tumorigenicity

Male and female Crl:NIH-*Foxn1*^*rnu*^ athymic nude rats (strain Code 316, Charles-River Laboratories) received CNS10-NPC-GDNF unilaterally to the lumbar spinal cord at 50,000 or 200,000 cells in µl, with 2 µl per injection administered into five sites providing a total dose of 0.5 × 10^6^ and 2 × 10^6^ cells, or vehicle. Experiments were performed at BTS Research in compliance with GMP standards in accordance with the US FDA GLP regulations, standards and guidelines (21 CFR Part 58). Sample size per sex was *n* = 6 for 30-d (±2) and *n* = 20 for 180-d (±3) timepoints post-operatively, with animals perfused with 4% paraformaldehyde and 4% sucrose in 0.1 M phosphate buffer followed by necropsy and tissue collection. Evaluations were performed blinded to treatment group.

### Cell preparation

CNS10-NPC-GDNF vials were thawed at 37 °C and cells were resuspended in transplantation medium supplemented with 2.6% DNase I (Pulmozyme, Genentech). Cells were centrifuged at 200*g*, 4 °C for 5 min. Supernatant was discarded and total cell yield was calculated using trypan blue exclusion. Cells were washed in transplantation medium, centrifuged and resuspended at the final cell concentration in transplantation medium.

### Cell delivery

Following a laminectomy and opening of the dura, CNS10-NPC-GDNF or vehicle was delivered via five unilateral injections (2 µl per site at a rate of 1 µl a min) into the lumbar spinal cord. A Hamilton syringe with a pulled glass micropipette was attached to a microinjector pump (World Precision Instruments) and stereotactic device (David Kopf Instruments). Coordinates were approximately medial/lateral = 0.75 from the midline, dorso/ventral = 1.65 from the dorsal surface of the spinal cord with 1.2 mm rostro/caudal intervals between injections. The micropipette was left in place for 2 min after cell infusion to reduce cell reflux up the injection tract.

### Locomotor rating scale

A modified BBB rating scale assessed motor function. Animals were placed in an open field and movements of the right and left hindlimbs were observed for ~4 min. Each limb was assigned a score between 0 (no observable movement) and 21 (consistent plantar stepping and coordinated gait, consistent toe clearance, predominant paw position is parallel throughout stance, consistent trunk stability, tail consistently up). Assessment was performed blinded to the treatment groups.

### Von Frey assay

An anesthesiometer (electronic Von Frey, IITC Life Sciences) was used for right and left hind paws. Each rat was individually placed in a measurement chamber positioned atop a metallic mesh grid. Following a short habituation period, supertip number 14 was then presented through the grid alternatively to the plantar aspect of each hind paw. As disease progressed, animals were less likely to withdraw their paws and particular attention was given to aversive reactions in response to the stimulus.

### Randall–Selitto assay

An analgesy-meter (Ugo Basile SRL) was used on the right and left hind paws. The meter exerts a mechanical force generated by a weight-displacing screw driven by a low-voltage geared motor, which provides an increase in force at a constant rate. The force is continuously monitored by the pointer moving over the linear scale. The rat was gently restrained and the hind paw was guided onto the small plinth under a cone-shaped pusher with a rounded tip. A force (0 to 250*g*) was applied by the meter to the hind paw. A pedal-switch exerted force at a constant rate of 16 g each s. When the rat elicited paw withdrawal or showed nocifensive behavior, the pedal was released and the applied force was recorded.

### Flinch–jump

A modified flinch–jump test was performed to assess electric footshock-evoked pain response. Footshock intensities (ranging from 0.2 to 1.0 mA) were presented through a floor bar-grid using the Freeze Monitor System (www.sandiegoinstruments.com). Rat response was rated by a blinded observer as flinch or jump or no response. The pain threshold (electric shock intensity at which jump was evoked) was measured through a maximum of three trials. This was performed only on the onset cohort because endpoint animals would be unable to maintain their bodies above the floor of testing chamber.

### Pig transplantation

Cell lines and protocols were used in accordance with guidelines approved by the SCRO and the IRB under the auspice of IRB-SCRO protocols 21505 and 29996. Pig work was performed following the guidelines of the Cedars-Sinai Medical Center IACUC under protocol number 3341. Naïve male and female Yucatan mini-pigs were injected with CNS10-NPC-GDNF at the following doses into ten sites: 20,000 cells (*n* = 3), 50,000 cells (*n* = 3) and 200,000 cells in 1 µl (*n* = 3), with injections in a 10 μl volume at 2-mm intervals. Cells were delivered directly into the ventral horn region because previous studies suggested that cell migration may be limited in the naïve CNS without signals of damage^19,49^. Triple immunosuppression therapy was a combination of Prograf (0.05 mg per kg each d) continuous infusion, mycophenolate mofetil (500 mg) twice daily and basiliximab (20 mg) intraoperatively and 4 d post-operatively. Pigs were killed at 29–31 d post-transplantation. Absorption Systems performed the GLP study under a protocol approved by their IACUC (number 15C302Q1R1), with 26 male and 26 female Yucatan mini-pigs receiving vehicle or CNS10-NPC-GDNF at the following doses into ten sites: 20,000 cells, 50,000 cells and 100,000 cells in 1 µl, with injections in a 10-μl volume at 2-mm intervals. Survival was 7 or 30 d, and each timepoint had *n* = 8 for the vehicle group and *n* = 6 per cell dose group.

### PNM score

Motor function was evaluated daily using a 14-point scale (PNM score). Animals were allowed to walk freely in an open space, and hind limb movement was observed for 5 min and assigned a score between 0 (no observable movement in either hind limb and tail) and 14 (capable of standing up spontaneously on hind limbs with sustained locomotion; consistent plantar-hoof stepping; consistent forelimb–hind limb coordination; able to pass hind limbs clearance test; tail movement present). This assessment was performed blinded to treatment groups.

### Rodent immunohistochemistry

For the dose ranging study, spinal cords were sectioned at 35 μm with a sliding microtome. For immunofluorescence, sections were washed with 1× PBS, blocked with Triton X-100 and normal donkey serum, incubated with primary antibodies overnight at room temperature (Supplementary Table 3), washed and incubated with secondary Alexa-488 or -594 antibodies (1:500; Thermo Fisher Scientific), washed and stained with nuclear counterstain 4′,6-diamidino-2-phenylindole (DAPI, Thermo Fisher Scientific), washed and cover-slipped. For GDNF, samples were washed with 1× PBS, quenched using 3% H_2_O_2_, washed, blocked in Triton X-100 with BSA and normal horse serum, incubated with primary antibody overnight at room temperature, washed, incubated with biotinylated secondary antibodies (Vector Laboratories), washed, incubated with avidin–biotin, washed and incubated with 3,3′-diaminobenzidine tetrahydrochloride (DAB, Vector Laboratories) and nickel substrate (Ni-DAB). For the GLP toxicity and tumorigenicity study, brain and spinal cord were processed by NeuroScience Associates. Samples were embedded in a gelatin matrix using multiCord or multiBrain technology, cured with a formaldehyde solution and rapidly frozen by immersion in chilled 2-methylbutane and sectioned on a microtome. Sections were H&E stained or processed for immunohistochemistry with antibodies to GDNF (1:150,000; BAF212, R&D Systems), human nestin (1:60,000; ABD69, Millipore), neurofilament (NF-1, 1:15,000; N2142, Sigma-Aldrich) and S100b (1:3,000,00; DAKO), followed by Ni-DAB. Tissue evaluations performed by a board-certified veterinary neuropathologist at Tox Path Specialists.

### Mini-pig immunohistochemistry

Sections were washed in 1× PBS, quenched in 3% H_2_O_2_, blocked in Triton X-100 with blocking serums (2.5% normal horse (STEM123, STEM121), 10% normal rabbit (GDNF), 10% normal goat (nestin)) and incubated with primary antibodies overnight at room temperature (Supplementary Table 3). Sections were washed and incubated in secondary biotinylated secondary antibodies (Vector Laboratories), and then washed and incubated in Vectastain ABC Elite Reagent (Vector Laboratories). For STEM121, STEM123 and nestin, ImmPACT NovaRED (Vector Laboratories) was used as the chromogen. For GDNF, Pierce metal-enhanced DAB (Thermo Fisher Scientific) was used as the chromogen. All used Mayer’s hematoxylin (Sigma) as the counterstain. In the GLP study, tissue was collected by Absorption Systems and processed by Inotiv (formerly, Seventh Wave) in a similar fashion to above.

### Cell counts

A blinded investigator used an optical fractionator method from Stereo Investigator software (MBF Biosciences) associated with an Axio Imager M2 microscope (Zeiss), with a ×40 apochromatic objective and a black and white camera. Contours delineating the region of interest were generated on the ipsilateral and contralateral sides of 1/24 sections. ChAT+ motor neurons were identified on the basis of correct anatomical location (ventral horn, laminae IX) and counted as positive only if associated with 4′,6-diamidino-2-phenylindole + nuclei. Alpha-motor neurons were defined as cells with an area >700 µm^2^. Nestin or GFAP-positive cells within the total cell population were determined by quantifying total number of Stem101-expressing cells and subsequently cells double labeled with Stem101 and human nestin or GFAP.

### Meningeal mass

Nissl-stained meningeal mass was scored on a qualitative scale from 0 to 4, with 0 indicating lack of dorsal mass and 4 indicating largest mass.

**Qualitative scale for meningeal mass**

**Table:** 

Grade	Description
0	Tissue is considered normal.
1	Severity grade slight. Microscopic change that barely exceeds what would be considered to be normal conditions. Typically, microscopic changes that are sporadic, focal and of no biologic significance to the function or structure of the tissue.
2	Severity grade minimal. Microscopic change that is more readily apparent in the tissue than a grade 1 change, but still unlikely to produce structural or functional impairment. Typically, microscopic changed are present in one or a few foci.
3	Severity grade moderate. Microscopic change that is prominent, conspicuous and an easily identified feature of the tissue, but still not present at considered maximum possible effect. Change would reasonably be expected to have some effect on the structure and/or function of a tissue although the correlating functional change may or may not be apparent.
4	Severity grade severe. Microscopic change that is prominent, conspicuous and an easily identified feature of the tissue, present at a maximum severity (affects majority of tissue and/or is very pronounced in one or more foci), and/or is present at a severity that would be expected to have a prominent effect on the structure and/or function of a tissue, although the correlating function change may or may not be apparent.

### GDNF enzyme-linked immunosorbent assay

Rat homogenates were prepared from ipsilateral spinal cord from wild-type and SOD1 animals at disease onset. For optimal comparison, pairwise comparisons for treatment and genotype interactions were adjusted for sex, with a Tukey adjustment. For participants, the ipsilateral-treated lumbar spinal cord was weighed and suspended in ice-cold homogenization buffer with protease inhibitor at a volume of 1 ml for every 10 mg of tissue. Tissues were homogenized with a Polytron mechanical dissociator for 30–40 s on ice, then centrifuged at 4 °C for 5 min. Supernatant containing the soluble fraction was transferred to 1.5-ml conical tubes and stored at −80 °C. A GDNF DuoSet enzyme-linked immunosorbent assay (ELISA) kit (DY212, R&D Systems) was used according to manufacturer’s instructions. Thawed soluble protein fractions were diluted in reagent diluent (two separate dilutions for each sample, with technical triplicates). Optical density was determined using a microplate reader, and GDNF protein concentration was normalized using the Bradford method.

### Cedars-Sinai injection device

Cedars-Sinai developed a new minimally invasive stereotactic delivery device and novel cannula. For ease of use, this device is a one-piece instrument not requiring assembly over the wound and attaches to a commercial minimal invasive retractor system. Compared with the Spinal Derrick Platform^11,22^, the Cedars-Sinai device has reduced incision size to only 4 cm compared with 10 cm, with improved stability, reduced procedural complexity and minimized risks by removing the need for percutaneous screws. The stereotactic device component mounts onto the retractor system (Medtronic MAST QUADRANT Retractor) after the laminectomy to provide access to the spinal cord. The cannula is inserted in a stereotactic device for cell delivery. It is a unique design that is ‘free floating’ and moves in the dorsal and ventral planes in response to movements of the spinal cord. The stereotactic device provides positioning through three-dimensional linear translation; angular adjustments are provided by the two primary axes. This permits accurate cannula positioning. The retractor and stereotactic device are multi-use; the cannula is a single-use device.

### Trial design

The trial was a phase 1/2a, single-center, safety study of two escalating doses of human neural progenitors producing GDNF (CNS10-NPC-GDNF) delivered unilaterally to the L3–L5 segment of the lumbar region of ambulatory patients with ALS with moderate leg involvement. Dose escalation reflects movement from the first dose cohort to the second dose cohort only after reviewing safety findings with the medical monitor. The Cedars-Sinai Office of Research Compliance and Quality Improvement (study IRB number Pro00042350) approved the study and participants signed the study informed consent form. Participants provided demographic information such as general health information, ethnicity, race, activity level, gender, age, height and weight. Participants, neurologists and study staff were blinded to injection side; however, the neurosurgeon was unblinded. The untreated limb served as a control. Participants were randomized to treatment side and enrolled into one of two dosing groups (the first and last participant enrolled on 20 April 2017 and 24 September 2018, respectively). Group A received 0.2 × 10^6^ cells in a 10-µl volume into ten sites and group B received 0.5 × 10^6^ cells in a 10-µl volume. Participants were followed for long-term vitality status and planned post-mortem tissue collection. The primary aim of this phase 1/2a trial is related to safety. The power calculations given in the statistical analysis plan specify the minimum rates of adverse events at any dosage that would be detected with high probability and the dosage-dependent difference in rates that would be detected with high probability. As binary outcomes (observance or not of a given type of adverse event), the variance is determined by the stated frequency (*p*) and sample size (*n*) (that is, variance = *np*(1 – *p*)).

### Cell product delivery

Vials of CNS10-NPC-GDNF p29 cells were thawed (City of Hope) at 37 ± 3 ˚C and washed with transplantation media supplemented with 2.6% Pulmozyme (26 μg ml^−1^; 889 nM). Cells were then centrifuged and combined into one suspension that was assessed for cell viability and concentration via trypan blue. The sample was washed with transplantation media (without Pulmozyme), centrifuged and resuspended in transplantation media at a volume equivalent to 65%–70% of the estimated final volume required. This suspension was counted for cell number and viability post-centrifugation, which was reported on the Certificate of Analysis. Based on total cell recovery, cells were resuspended in an appropriate volume of transplantation media. The final dose was packaged in a CryoElite Vial (Wheaton) and shipped to Cedars-Sinai Investigational Pharmacy in a GTS-77 Shipping System (Cold Chain Technologies), where cells were transferred to a 2–8 °C storage unit. Within 24 h, the surgical suite requested the Investigational Pharmacy to deliver cells, which were loaded into the cannula under an aseptic protocol. A Mayo stand and the Tritech MINJ-PD pump were covered with sterile drapes, and the cannula and Hamilton syringe were aseptically removed from the sterile packaging and placed on the sterile field. The cannula was then primed with 0.9% saline until a drop of saline exited the tip. The cells from the vial were resuspended and loaded into the cannula for delivery at a rate of 5 μl each min for a total of 10 μl per site. The side treated was randomly assigned in a 1:1 ratio using a computer-generated with permuted blocks, stratified by cohort.

### Immunosuppression and neuropathic pain medication

Basiliximab (Simulect, Novartis) 20 mg was administered intravenously at surgery. A second dose of 20 mg was given on post-operative day 4. Tacrolimus (Prograf; Fujisawa Healthcare) was started on transplant day at doses of 1–6 mg each d divided every 12 h adjusted to maintain trough levels of 4–8 ng per ml. Trough levels were measured as necessary to maintain the target range. Targeted trough levels were: 4–8 ng per ml for months 0–3; 4–7 mg per ml for months 3–6; 4–6 ng per ml for months 6–9; and 4–5 ng per ml for months 9–12. Trough levels were checked 2 weeks after any dose change. Mycophenolate mofetil (Cellcept) was administered starting on post-transplant day 0 at 0.5 g twice a day. Methyprednisolone (125 mg, intravenously) was given intraoperatively, followed by a taper of oral prednisone 60 mg daily on post-operative days 1–4, 40 mg daily on post-operative days 5–8, 20 mg daily on post-operative days 9–16, 10 mg daily on post-operative days 17–21, and 5 mg daily on post-operative days 22–28. At 28 d, prednisone was discontinued. Neuropathic pain was primarily managed with gabapentin (100–1,800 mg daily) and in some instances analgesics.

### MRI

Participants had preoperative thoracolumbar MRI scanning in a Siemens Skyra 3T magnet. Unenhanced spine MRI included the following sequences: sagittal T1 turbo spin-echo (tse) 2 mm, sagittal T2 tse 2 mm, sagittal T2 space 1 mm, coronal T2 space 1 mm, coronal T1 tse 2 mm and axial T2 tse. For post-operative imaging, contrast-enhanced MRI with sagittal and axial T1 sequences following intravenous Gadovist (gadobutrol, 1.0 mmol per ml, a nonionic macrocyclic agent; Bayer Shering Pharma) were added to the unenhanced protocol.

### Quantitative muscle strength testing

ATLIS is a quantitative strength assessment system utilizing a fixed frame and mounted force plate^52,53^. Absolute isometric strength is assessed for each leg when performing three maneuvers: knee extension, knee flexion and ankle dorsiflexion. Knee flexion and extension were tested with the participant seated upright in testing chair with the knee flexed at 90°. Ankle dorsiflexion was tested with the load cell just above the dorsum of the foot, proximal to the toes. The load cell transmitted a wireless signal to a computer that displayed real-time force curves and recorded the peak force. Each maneuver was performed twice. A third trial was performed if the force recorded for the first two trials differed by more than 15%. If a participant could not complete a maneuver because of weakness, a force of zero was scored. A lack of force recordings for any other reason was treated as missing. The maximum strength across all trials for a given maneuver for a given leg at a given visit was used for analysis. ATLIS testing took ~15 min. Force in pounds was converted to percent of predicted normal based on each participant’s sex at birth, age at the time of testing, baseline weight and baseline height using published norms, including updates for knee extension using the ATLIS 2.0 device^52,53^. When ATLIS was unavailable at a 3-year post-transplantation evaluation for participant 113, muscle strength was measured using the Medical Research Council scale, which is determined by clinician-administered manual muscle testing and scored on a scale of 0–5, with a score of 5 indicating normal strength and a score of 0 indicating no volitional movement of the muscle is possible. Deep tendon reflexes were also assessed in the upper and lower extremities bilaterally, and the response was graded on a scale of 0–4+, with 2+ considered normal.

### Secondary outcome measures

The ALSFRS-R is a patient-reported, clinician-assessed, 48-point scale that measures overall functional status. Other functional testing used to compare innervation of the treated and untreated leg included CMAP of the tibialis anterior and EIM. Supramaximal CMAP was recorded from the tibialis anterior with stimulation of the fibular nerve at the fibular head. The active electrode was placed on the muscle belly ~8 cm inferior to the tibial tuberosity with the reference electrode placed distally along the tibialis anterior tendon and the ground electrode placed between the active electrode and the fibular head. EIM was performed using the Myolex mView EIM system by placing the measuring device directly on the skin over the knee extensors (quadriceps), ankle plantarflexors (gastrocnemius) and ankle dorsiflexors (tibialis anterior), and measurements obtained from the 50 kHz phase were recorded.

### Post-mortem tissue collection

Consent for autopsy and tissue collection was obtained from next of kin at the time of participant death. Brain and spinal cord were harvested within less than 24 h (4–18 h) of time of death. Following spinal cord dissection, the transplant region was isolated, and a ~2 mm block of spinal cord was paraffin-embedded, sectioned at 5 μM and sent for histopathological analysis by a board-certified pathologist. The remaining spinal cord was cryoprotected in 30% sucrose and cut into 35-µm sections using a microtome.

### Nested PCR

Primers were designed to target the mPGK promoter and human GDNF sequences of the pSIN-WP-mPGK-GDNF lentiviral construct to yield a PCR product unique to CNS10-NPC-GDNF. Primers that amplify a segment of the human MMP28 gene were used to confirm the presence of human genomic DNA in all samples. Total genomic DNA was extracted from four to eight formalin-fixed tissue sections following the manufacturer’s instructions (DNeasy Blood & Tissue Kit, Qiagen), with the modification that following the initial 1-h Proteinase K digestion an additional 20 μl of Proteinase K was added and incubation at 56 °C continued until tissue fragments were no longer observed (typically 20 min). DNA was eluted using 100 μl of the provided elution buffer. Because of a low signal for engrafted CNS10-NPC-GDNF in the tissue section, total human genomic DNA was subject to two rounds of amplification utilizing a nested PCR approach. MyTaq HS Red Mix was used to perform the amplification following manufacturer’s instructions (Bioline). Thermal amplification conditions were as follows: 95 °C for 5 min of heat denaturation followed by 35 cycles of annealing at 60 °C for 20 s, extension at 72 °C for 20 s and denaturation at 95 °C for 20 s. To limit the nonspecific background observed in a few cases, a 62 °C annealing temperature was used. Amplified DNA was visualized following agarose gel electrophoresis utilizing a Bio Rad Gel Doc XR imager. Complete gels provided in Source Data Fig. 1.

**PCR primers**

**Table:** 

pgk-GDNF Round 1	PGK-GDNF-F1	CTTTCTGGGCTCAGAGGCT	447 bp
	PGK-GDNF-R1	TATCTGGTGACCTTTTCA	
pgk-GDNF Round 2	PGK-GDNF-F2	GGGTCCGGGGGCGGGCTC	200 bp
	PGK-GDNF-R2	GCCACGACATCCCATAAC	
Genomic DNA Control Round 1	MMP28_PCR-F1	GGAAATCTTGGCCTAGCCGG	444 bp
	MMP28_PCR-R1	TGAACAGGTCCCCAAAGCTCC	
Genomic DNA Control Round 2	MMP28_PCR-F2	TAACAGTGCTCACCTTGC	398 bp
	MMP28_PCR-R2	ACCTCCACTCGATTCAGC	

### Immunohistochemistry

Sections were washed three times in PBS for 5 min, quenched in 0.3% H_2_O_2_ for 30 min, washed in 0.05% Triton X-100 in 1× PBS (PBS-T), then blocked in 3% normal horse serum and 2% BSA (Sigma-Aldrich) for 60 min at room temperature, followed by incubation in primary antibody at 4 °C, except for GDNF which was at room temperature (Supplementary Table 3). Sections were then washed with 0.2% PBS-T and placed in appropriate biotinylated secondary antibodies (Vector, BA-9500 or BA-1100, 1:200) for 2 h at room temperature. After washing, sections were incubated in avidin–biotin complex (Vector, PK-4000) for 45 min at room temperature and washed for 30 min in 1× PBS. A DAB kit (Vector, SK-4100) was used for chromogenic detection. For GDNF staining, samples were enhanced with nickel substrate coincubation. Sections were then washed in tap water and 1× PBS, mounted on slides and dehydrated in two washes each of 95% ethanol (EtOH), 100% EtOH and xylene. Images taken with Leica DM 2000 LED microscope using Leica ICC50 HD camera.

### Histology

For regressive H&E staining, dried mounted sections were defatted by 2-min rinses in 70% EtOH, 100% EtOH, xylene, 100% EtOH, 70% EtOH and 20% EtOH, then washed for 1 min in distilled water (dH_2_O). Sections were stained with hematoxylin for 10 min and washed for 1.5 min in tap water. Slides were dipped between five and ten times in 1% HCl in 70% EtOH, washed in tap water, dipped for 30 s in 0.5% lithium carbonate (Rowley Biochemicals, K-680-1) in 0.1% ammonia water (Electron Microscopy Sciences, 26698-02), washed in tap water and stained with eosin (Sigma-Aldrich, HT110332) for 5–10 s. Slides were dehydrated for 3 min each in 95% EtOH, three changes of 100% EtOH and three changes of xylene before being cover-slipped with permanent mounting media (Thermo Scientific, 4112). For Nissl staining, dried mounted sections were rehydrated in dH_2_O for 5 min, submerged for 5 min in 70% EtOH, washed for 5 min in dH_2_O and dipped for 3 min in 0.5% cresyl violet (Sigma-Aldrich, C5042-10G) in dH_2_O. Slides were dipped three times in dH_2_O followed by a differentiation solution of 10% acetic acid in 70% EtOH for six dips, and a differentiation solution of 10% acetic acid in 100% EtOH for six dips. Slides were dehydrated for 2 min in 100% EtOH, then for 5 min in xylene and cover-slipped with permanent mounting media. Images were taken with Leica DM 2000 LED microscope using a Leica ICC50 HD camera.

### Motor neuron counts

Every 12th section was imaged on a Zeiss Axio Imager 2. Tiled images (×10) were stitched together using a Zeiss Zen microscope. Using stereology and performed blinded, images were counted for ChAT+ motor neurons. For participant 113, every 12th section was imaged on a Leica DM 2000 LED microscope using a Leica ICC50 HD camera and the Leica Application Suite EZ Program (v.3.4.0). Images magnified at ×5 were then counted for ChAT+ motor neurons with intact axon and soma morphology on ImageJ using the Freehand selections tool and the region of interest manager.

### Statistical analysis

#### Animal studies

A senior statistician from the Cedars-Sinai Biostatistics and Bioinformatics Research Center performed or reviewed the statistical analyses. Software used was SAS v.9.4, GraphPad or Asreml 3.0 in R 3.14 × 64 (VSN international). All testing was considered statistically different at the two-sided level of *P* < 0.05. Error terms are presented as standard error mean (s.e.m). Any categorical data were tested with a chi-squared test. For continuous data measurements, model residuals were inspected to confirm that data met the assumptions for parametric analysis. Where data failed to meet the assumptions necessary for parametric assessment, continuous data were transformed before analysis by way of the Box–Cox transformation. Because no differences were observed between the sexes, data for males and females were combined. The Tukey method was used to adjust for multiple comparisons. For the locomotor rating scale, the difference in absolute BBB scores between limbs for each animal was computed as the score for the ipsilateral minus the contralateral. For the locomotor rating scale and Von Frey and Randall Selitto assays, differences between treatment group means were tested over time in a generalized mixed-model regression analysis using compound symmetry covariance matrix. For the grafted cell phenotype and GDNF production (ELISA) assays, differences between groups were tested with a general linear regression model. For motor neuron counts, the treated and untreated sides were compared across groups. Counts were normalized for the variance between animals by taking the ratio of >700 µm^2^ cells against total ChAT-positive cells. This normalized count was used as the dependent variable for statistical analyses using a mixed-model regression with compound symmetry covariance structure, and was Tukey-adjusted for multiple comparisons. Differences were considered significant at the two-sided level of *P* < 0.05.

#### ATLIS

The effect of CNS10-NPC-GDNF treatment versus no treatment on average leg strength assessed by ATLIS was tested using a shared-baseline mixed model. The model included fixed effects for dosage cohort (two levels: low = 2 × 10^6^ cells, high = 5 × 10^6^ cells); leg side (two levels: left, right), visit (six levels: baseline and months 1, 3, 6, 9 and 12); all two- and three-way interactions of dosage cohort, leg side and visit and the four-way interaction of dosage cohort; injection side (two levels: ipsilateral, contralateral); leg side and post-baseline visit (five levels). This construction specifies an unstructured cell-means model other than the assumption of a shared baseline before randomization. The model included random intercepts and slopes at the level of participant and at the level of leg side nested within participant, each with unstructured covariance. The interaction between fixed effects for injection side and visit was restricted to post-baseline visits by including a numeric indicator variable (0 pretreatment, 1 post-treatment) in the interaction. Use of a shared baseline reflects the true state of the population sampled before randomization and has the advantage of adjusting for any chance differences at baseline in a manner similar to analysis of covariance^54^. Although separate modeling of leg side (left versus right) and injection side (ipsilateral versus contralateral) over-specifies the model, this construction accommodates the tendency for left legs to be stronger while also permitting direct estimation of the contrast in strength between legs on the treated versus untreated side. The primary estimator was the difference in slope of average leg strength from baseline to month 12 between the treated and untreated leg, averaged across dosage cohorts and tested using a two-tailed Wald-test at alpha = 0.05. The estimate and its 95% confidence bounds were obtained by a linear contrast of visit-specific adjusted means with coefficients of −31, −25, −13, 5, 23 and 41, and a divisor of 665 for the treatment side-dependent differences at months 0, 1, 3, 6, 9 and 12, respectively, to yield an estimate in units of change per month given the visit times.

#### Participant motor neurons

A senior statistician from the Cedars-Sinai Biostatistics and Bioinformatics Research Center performed the statistical analysis. Differences across treated and untreated sides in ChAT cell counts were tested with mixed Poisson regression. All modeling included correlated observations with hierarchical linear modeling with the fixed effect of transplant side, and random within-subject effects. Given the presence of high outliers, area data were log-transformed before analysis to meet the assumptions necessary for linear regression. Data were considered statistically significant where *P* < 0.05. Statistical analysis performed using SAS v. 9.4 software.

### Reporting summary

Further information on research design is available in the Nature Research Reporting Summary linked to this article.

## Online content

Any methods, additional references, Nature Research reporting summaries, source data, extended data, supplementary information, acknowledgements, peer review information; details of author contributions and competing interests; and statements of data and code availability are available at 10.1038/s41591-022-01956-3.

## Supplementary information

Supplementary Information Supplementray Tables 1–3.

Reporting Summary

Supplementary Video 1 Participant 113 at 3 years post-transplantation.

## Data Availability

All requests for raw and analyzed data and materials are promptly reviewed by the Cedars-Sinai Board of Governors Institute of Regenerative Medicine to verify if the request is subject to any intellectual property or confidentiality obligations. Patient-related data not included in the paper were generated as part of clinical trials and may be subject to patient confidentiality. Any data and materials that can be shared will be released via a Material Transfer Agreement. All raw and analyzed sequencing data can be found at the NCBI Sequence Read Archive (accession number: ON053114). Source data are provided with this paper.

## Source data

Source Data Fig. 3 Gels from Fig. 3d with ladders.
