# Supplementary material for: Transplantation of human neural progenitor cells secreting GDNF into the spinal cord of patients with ALS: a phase 1/2a trial
Source: Nat Med. 2022 Sep 5;28(9):1813–22. doi: 10.1038/s41591-022-01956-3 (PMC9499868; doi:10.1038/s41591-022-01956-3)
Supplement: Supplementary file 1 — Supplementray Tables 1–3. [file 41591_2022_1956_MOESM1_ESM.pdf]

nature portfolio

https://doi.org/10.1038/s41591-022-01956-3

Supplementary information

Transplantation of human neural progenitor cells secreting GDNF into the spinal cord of patients with ALS: a phase 1/2a trial

In the format provided by the authors and unedited

Subject	Side	Description	Severity	Outcome*	Start (Post-op Day)	Stop (Post-op day)
101	Treated	Leg Pain - Needles	8	Change in Severity	0	3
	Treated	Leg Pain - Needles	3	Ongoing	3	-
	Bilateral	Leg Pain - Aching	5	Ongoing	594	-
102	Treated	Foot Numbness/Tingling	2	Change in Severity	0	170
	Treated	Leg Numbness/Tingling	5	Resolved	170	215
	Treated	Foot Numbness	Moderate	Resolved	160	215
	Untreated	Leg Pain - Dull	2	Resolved	179	179
	Bilateral	Leg Pain - Dull	3	Ongoing	335	-
103	Treated	Knee Pain (Intermittent) - Sharp	4	Ongoing	95	-
	Treated	Leg Soreness	4	Ongoing	40	-
	Treated	Leg Pain - Burning	7	Change in Severity	246	338
	Treated	Leg Pain - Dull Burning	3	Ongoing	338	-
104	Treated	Leg Pain - Aching	7	Change in Severity	0	1
	Treated	Leg Pain - Aching	3	Change in Severity	1	3
	Treated	Leg Pain - Aching	6	Resolved	3	152
	Treated	Foot Pain (intermittent) - Aching	6	Resolved	1	61
	Untreated	Foot Pain - Sharp	8	Resolved	68	319
105	Treated	Thigh Pain - Pins and needles	5	Change in Severity	26	47
	Treated	Thigh Pain - Pins and needles	2	Ongoing	47	-
106	Treated	Leg Pain - Aching	7	Resolved	0	2
	Bilateral	Leg Pain - Shooting	4	Resolved	8	11
	Treated	Leg Pain - Stabbing/Burning	6	Resolved	214	275
	Bilateral	Leg - Loss of pin sensation	Mild	Ongoing	180	-
107	Treated	Calf Pain - Shooting	5	Resolved	0	5
	Treated	Knee Pain - Shooting	8	Resolved	0	5
	Treated	Foot Pain - Shooting	8	Resolved	0	7
	Bilateral	Leg Pain - Bee Stings/Pin Pricks	7	Change in Severity	55	58
	Bilateral	Leg Pain - Sharp	4	Change in Severity	58	91
	Bilateral	Leg Pain - Ant bites	9	Change in Severity	91	91
	Bilateral	Leg Pain - Ant bites	3	Resolved	91	113
	Treated	Leg - Decreased pin sensation	Mild	Resolved	33	92
108	Untreated	Leg Pain - Aching	5	Resolved	47	77
109	Treated	Leg Pain - Stabbing	5	Resolved	0	7
	Bilateral	Calf Cramping (Intermittent)	8	Ongoing	245	-
	Bilateral	Leg Soreness	3	Ongoing	337	-
	Untreated	Leg Itching (Intermittent)	Moderate	Resolved	28	43
	Treated	Leg Itching (Intermittent)	Severe	Change in Severity	28	43
	Treated	Leg Itching (Intermittent)	Mild	Resolved	43	184
110	Treated	Thigh Pain - Shooting	5	Resolved	0	8
	Treated	Thigh Tingling	5	Resolved	0	8
	Treated	Lower Leg Pain - Pins and Needles	5	Ongoing	5	-
	Bilateral	Cramping	7	Ongoing	95	-
	Bilateral	Leg - Decreased pin sensation	Mild	Resolved	2	265
111	Untreated	Radiating when laying Down	2	Resolved	222	371
	Treated	Radiating when laying Down	2	Resolved	222	371
	Treated	Radiating warmth from hip to knee	3	Ongoing	19	-
	Bilateral	Leg - Decreased pin sensation	Mild	Ongoing	34	-
112	Treated	Lower Leg Tingling	5	Resolved	0	3
113	Treated	Leg Pain - Shooting/Tingling	5	Ongoing	0	-
	Untreated	Leg - Stabbing	8	Ongoing	18	-
114	Treated	Thigh Pain - Shooting	8	Change in Severity	1	32
	Treated	Thigh Pain - Shooting	9	Change in Severity	32	58
	Treated	Thigh Pain - Shooting	8	Change in Severity	58	94
	Treated	Thigh Pain - Shooting	3	Change in Severity	94	208
	Treated	Thigh Pain - Shooting	7	Ongoing	208	-
	Treated	Leg Pain - Aching	9	Ongoing	185	-
	Treated	Thigh Itching	Moderate	Ongoing	2	-
	Treated	Thigh Hyperesthesia	Moderate	Change in Severity	2	58
	Treated	Thigh Hyperesthesia	Mild	Ongoing	58	-
	Bilateral	Leg - Decreased pin sensation	Mild	Ongoing	33	-
115	Treated	Leg Pain - Burning	7	Resolved	0	504
116	Untreated	Leg Pain - Aching	2	Resolved	0	2
	Treated	Leg - Decreased pin sensation	Mild	Resolved	40	61
118	Treated	Foot Pain (intermttent)-Burning/Tingling	1	Resolved	1	99

***Resolved** = Resolved without sequela(e), **Ongoing** = Condition still present as of last contact.

9/18 participants had lower extremity pain on the treated side lasting longer than 6 months, of which 5 had pain ≥5 in severity

Supp Table 1. Detailed sensory changes reported by participants throughout the study.

Subject	Gabapentin Dose	Start (Post-op Day)	End (Post-op Day)
101	-	-	-
102	300mg	5	91
103	600mg	-1274	0
	1800mg	0	1
	1200mg	1	Ongoing
104	900mg	0	33
	300mg	62	450
105	900mg	1	5
	300mg	180	182
	600mg	182	184
	900mg	184	195
106	300mg	0	5
107	900mg	1	5
	300mg	63	82
	300mg	263	Ongoing
108	600mg*	0	0
109	900mg	1	21
	900mg	43	Ongoing
110	600mg	1	5
	600mg	43	Ongoing
111	-	-	-
112	100mg	331	Ongoing
113	600mg	0	5
	600mg	11	Ongoing
114	1200mg	63	Ongoing
115	1200mg	0	.
116	600mg*	0	0
117	-	-	-
118	800mg*	0	0
	600mg	2	5

*Single Dose

Supp Table 2. Dosage and duration of Gabapentin use by participants in the trial.

Primary antibodies for dose ranging and pig studies

Antibody[1]	Dilution Rats	Dilution Pigs	Company	Catalogue #
Human nuclei (Stem101)	1:200	Not used	Takara	Y40400
Human cytoplasm (Stem121)	1:2000	1:750	Takara	Y40410
Human nestin	1:15,000	1:8000	EMD Millipore	ABD69
GFAP	1:500	Not used	Dako	Z0334
Human GFAP (Stem 123)	Not used	1:1500	Takara	Y40420
ChAT	1:200	Not used	EMD Millipore	AB144P
GDNF	1:250	1:200	R&D Systems	BAF212
AQP4	1:100	Not used	Sigma	HPA014784
Glast	1:20	Not used	R&D Systems	AF6048

[1]Incubated overnight at room temperature

Primary antibodies for human spinal cord

Primary Antibody[1]	Dilution	Duration	Company	Catalog #
Human GDNF (biotinylated)	1:50	24 hours	R&D Systems	BAF212
Human-specific ChAT	1:200	48–72 hours	R&D Systems	AF3447
IBA1	1:250	Overnight	Novus Bio	NB100-1028
Human-specific Nestin	1:2000	Overnight	EMD Millipore	ABD69
GFAP	1:500	Overnight	Dako	Z0334
Ki-67	1:200	Overnight	Thermo Scientific	RM-9106-S
CD34	1:500	Overnight	Abnova	PAB18289
Collagen IV	1:150	Overnight	Rockland	600-401-106-0.1
S100b	1:100	Overnight	Sigma-Aldrich	HPA015768
Human-specific NF-H	1:4000	Overnight	R&D Systems	AF3108

[1]Incubated at 4°C, except GDNF at room temperature

Supp Table 3. Antibody details
