# Supplementary material for: Transplantation of human neural progenitor cells secreting GDNF into the spinal cord of patients with ALS: a phase 1/2a trial
Source: Nat Med. 2022 Sep 5;28(9):1813–22. doi: 10.1038/s41591-022-01956-3 (PMC9499868; doi:10.1038/s41591-022-01956-3)
Supplement: Supplementary file 2 — Reporting Summary [file 41591_2022_1956_MOESM2_ESM.pdf]

nature portfolio

Corresponding author(s): Clive N. Svendsen

Last updated by author(s): May 13, 2022

Reporting Summary

Nature Portfolio wishes to improve the reproducibility of the work that we publish. This form provides structure for consistency and transparency in reporting. For further information on Nature Portfolio policies, see our Editorial Policies and the Editorial Policy Checklist.

Statistics

For all statistical analyses, confirm that the following items are present in the figure legend, table legend, main text, or Methods section.

n/a	Confirmed	
☐	☒	The exact sample size (n) for each experimental group/condition, given as a discrete number and unit of measurement
☐	☒	A statement on whether measurements were taken from distinct samples or whether the same sample was measured repeatedly
☐	☒	The statistical test(s) used AND whether they are one- or two-sided<br>*Only common tests should be described solely by name; describe more complex techniques in the Methods section.*
☒	☐	A description of all covariates tested
☐	☒	A description of any assumptions or corrections, such as tests of normality and adjustment for multiple comparisons
☐	☒	A full description of the statistical parameters including central tendency (e.g. means) or other basic estimates (e.g. regression coefficient) AND variation (e.g. standard deviation) or associated estimates of uncertainty (e.g. confidence intervals)
☐	☒	For null hypothesis testing, the test statistic (e.g. F, t, r) with confidence intervals, effect sizes, degrees of freedom and P value noted<br>*Give P values as exact values whenever suitable.*
☒	☐	For Bayesian analysis, information on the choice of priors and Markov chain Monte Carlo settings
☐	☒	For hierarchical and complex designs, identification of the appropriate level for tests and full reporting of outcomes
☐	☒	Estimates of effect sizes (e.g. Cohen's d, Pearson's r), indicating how they were calculated

Our web collection on statistics for biologists contains articles on many of the points above.

Software and code

Policy information about availability of computer code

Data collection	PharmaEngine EDC software.
Data analysis	All statistical analyses will be performed using SAS (SAS Institute, NC, USA) or R (R Foundation for Statistical Computing, Vienna, Austria).

For manuscripts utilizing custom algorithms or software that are central to the research but not yet described in published literature, software must be made available to editors and reviewers. We strongly encourage code deposition in a community repository (e.g. GitHub). See the Nature Portfolio guidelines for submitting code & software for further information.

Data

Policy information about availability of data

All manuscripts must include a data availability statement. This statement should provide the following information, where applicable:

- Accession codes, unique identifiers, or web links for publicly available datasets
- A description of any restrictions on data availability
- For clinical datasets or third party data, please ensure that the statement adheres to our policy

All requests for raw and analyzed data and materials are promptly reviewed by the Cedars-Sinai Board of Governors Institute of Regenerative Medicine to verify if the request is subject to any intellectual property or confidentiality obligations. Patient-related data not included in the paper were generated as part of clinical trials and may be subject to patient confidentiality. Any data and materials that can be shared will be released via a Material Transfer Agreement. All raw and analyzed sequencing data can be found at the NCBI Sequence Read Archive (accession number: pending).

Field-specific reporting

Please select the one below that is the best fit for your research. If you are not sure, read the appropriate sections before making your selection.

☒ Life sciences ☐ Behavioural & social sciences ☐ Ecological, evolutionary & environmental sciences

For a reference copy of the document with all sections, see nature.com/documents/nr-reporting-summary-flat.pdf

Life sciences study design

All studies must disclose on these points even when the disclosure is negative.

Sample size	Detailed in methods section
Data exclusions	Detailed in methods section
Replication	Detailed in methods section
Randomization	Detailed in methods section
Blinding	Detailed in methods section

Reporting for specific materials, systems and methods

We require information from authors about some types of materials, experimental systems and methods used in many studies. Here, indicate whether each material, system or method listed is relevant to your study. If you are not sure if a list item applies to your research, read the appropriate section before selecting a response.

Materials & experimental systems

n/a	Involved in the study	
☐	☒	Antibodies
☐	☒	Eukaryotic cell lines
☒	☐	Palaeontology and archaeology
☐	☒	Animals and other organisms
☐	☒	Human research participants
☐	☒	Clinical data
☒	☐	Dual use research of concern

Methods

n/a	Involved in the study	
☒	☐	ChIP-seq
☒	☐	Flow cytometry
☐	☒	MRI-based neuroimaging

Antibodies

Antibodies used

Human nuclei (Stem101) 1:200 ab-101-u-050 Takara Bio
Human cytoplasm (Stem121) 1:2000 ab-121-u-050 Takara Bio
Human-specific Nestin 1:15,000 ABD69 EMD Millipore
GFAP 1:500 Z0334 Dako
ChAT 1:200 AB144P-1ML EMD Millipore
GDNF 1:250 BAF212 R&D Systems
AQP4 1:100 HPA014784 Sigma
Glast 1:20 AF6048 R&D Systems
Human-specific GDNF (biotinylated) 1:50 BAF212 R&D Systems
Human-specific ChAT 1:200 AF3447 R&D Systems
Iba-1 1:250 NB100-1028 Novus Bio
Human-specific Nestin 1:2000 ABD69 EMD Millipore
GFAP 1:500 Z0334 Dako
Ki-67 1:200 RM-9106-S Thermo Scientific
CD34 1:500 PAB18289 Abnova
Collagen IV 1:150 600-401-106-0.1 Rockland
S100B 1:100 HPA015768 Sigma-Aldrich
Human-specific NF-H 1:4000 AF3108 R&D Systems

Validation

Antibodies were validated and optimized based on the manufacturer's recommendations and using known positive controls.

Eukaryotic cell lines

Policy information about cell lines

Cell line source(s)	Tissue Donation obtained by Dr. Guido Nikkah
Authentication	HLA typing was performed for the line at multiple timepoints during manufacturing for authentication.
Mycoplasma contamination	Cell lines are negative for mycoplasma and all adventitious testing
Commonly misidentified lines (See ICLAC register)	N/A

Animals and other organisms

Policy information about studies involving animals; ARRIVE guidelines recommended for reporting animal research

Laboratory animals	Male and female SOD1G93A transgenic rats (NTac:SDTg (SOD1G93A) L26H) and wild-type littermates were acquired from Taconics (Hudson, NY, USA) and maintained as a colony by in-house breeding with Sprague-Dawley females (Taconics), Age ~100 days at study start.<br>Female and Male Yucatan Mini Pigs, 14-20Kg at study start.
Wild animals	N/A
Field-collected samples	N/A
Ethics oversight	Detailed in methods section

Note that full information on the approval of the study protocol must also be provided in the manuscript.

Human research participants

Policy information about studies involving human research participants

Population characteristics	Main table 1
Recruitment	Detailed in methods section
Ethics oversight	Cedars-Sinai Office of Research Compliance and Quality Improvement - Study IRB# Pro00042350

Note that full information on the approval of the study protocol must also be provided in the manuscript.

Clinical data

Policy information about clinical studies

All manuscripts should comply with the ICMJE guidelines for publication of clinical research and a completed CONSORT checklist must be included with all submissions.

Clinical trial registration	NCT02943850
Study protocol	Requests can be made to, and promptly reviewed by, the Cedars-Sinai Board of Governors Institute of Regenerative Medicine
Data collection	Detailed in methods section and/or results section
Outcomes	Detailed in methods section and/or results section

Magnetic resonance imaging

Experimental design

Design type	Longitudinal
Design specifications	Participants were imaged at different timepoints for safety evaluations
Behavioral performance measures	Not Used

Acquisition

Imaging type(s)	Structural
Field strength	3 Tesla
Sequence & imaging parameters	Preoperative thoracolumbar MR in a Siemens Skyra 3T Magnet. The unenhanced spine MR included the following sequences: sagittal T1 turbo spin-echo(tse) 2mm, sagittal T2 tse 2mm, sagittal T2 space 1mm, coronal T2 space 1mm, coronal T1 tse 2mm, axial T2 tse. For postoperative imaging, contrast enhanced MR with sagittal and axial T1 sequences following intravenous Gadovist (gadobutrol, 1.0mmol/mL, a nonionic macrocyclic agent; Bayer Shering Pharma) were added to the unenhanced protocol.
Area of acquisition	Thoracolumbar

Diffusion MRI ☐ Used ☒ Not used

Preprocessing

Preprocessing software	Not used
Normalization	Not used
Normalization template	Not used
Noise and artifact removal	Not used
Volume censoring	Not used

Statistical modeling & inference

Model type and settings	Not used
Effect(s) tested	Not used

Specify type of analysis: ☐ Whole brain ☐ ROI-based ☐ Both

Statistic type for inference (See Eklund et al. 2016)	Not used
Correction	Not used

Models & analysis

n/a	Involved in the study
☒	☐ Functional and/or effective connectivity
☒	☐ Graph analysis
☒	☐ Multivariate modeling or predictive analysis
