# Supplementary figures and images for: Transplantation of human neural progenitor cells secreting GDNF into the spinal cord of patients with ALS: a phase 1/2a trial
Source: Nat Med. 2022 Sep 5;28(9):1813–22. doi: 10.1038/s41591-022-01956-3 (PMC9499868; doi:10.1038/s41591-022-01956-3)

ALS101

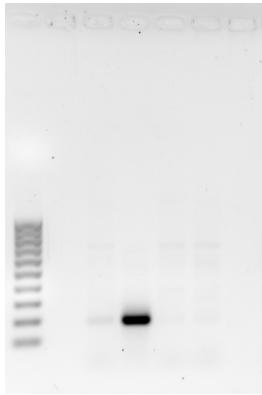

ALS102

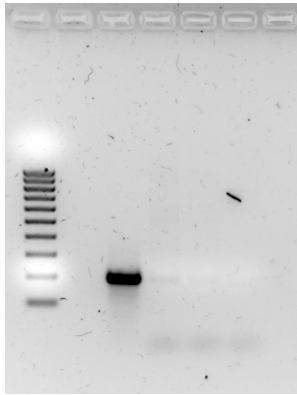

ALS103

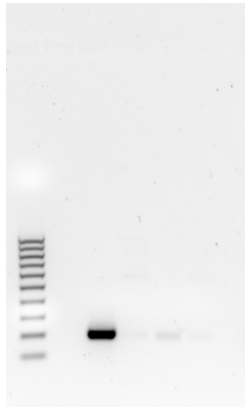

ALS113

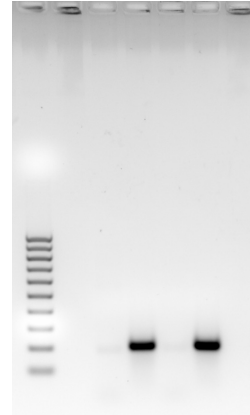

ALS104

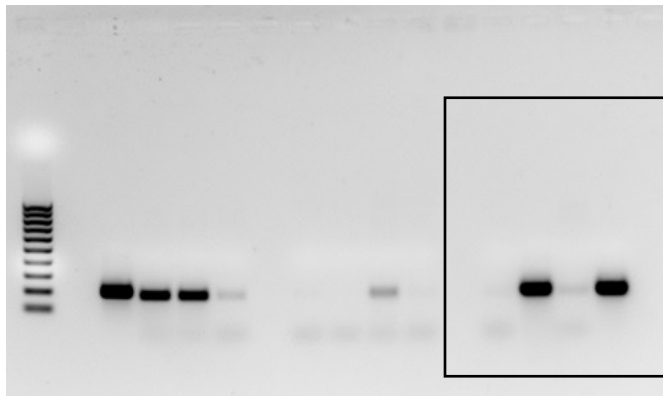

ALS105

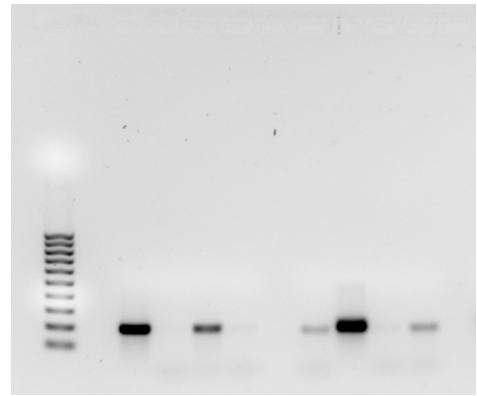

ALS106

ALS107

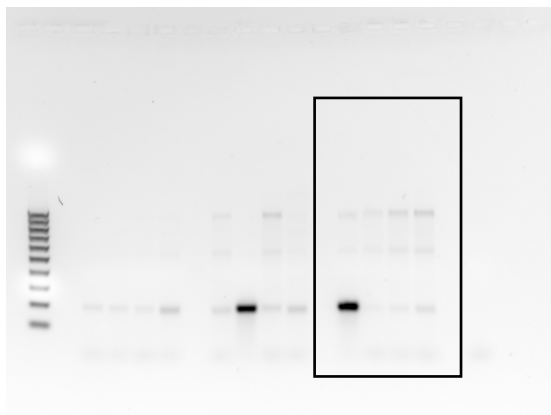

ALS109

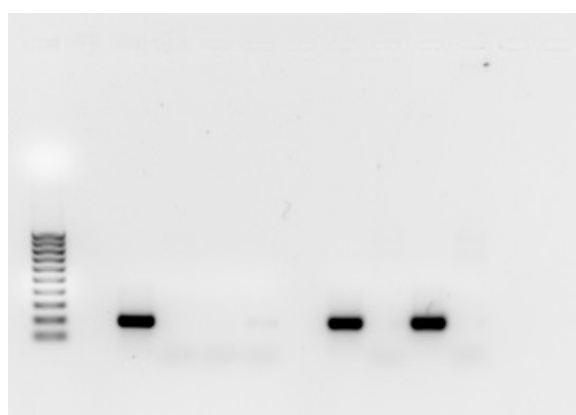

ALS111

ALS114

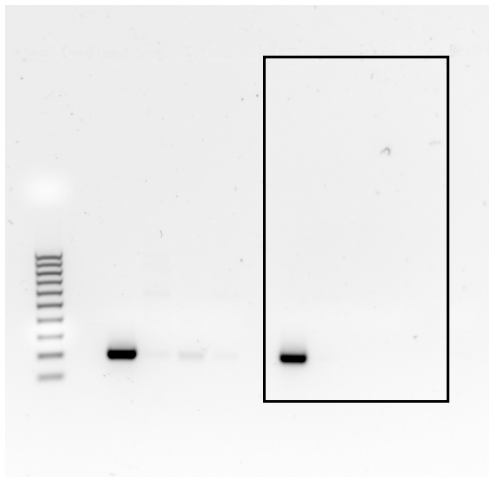Non-ALS  
Control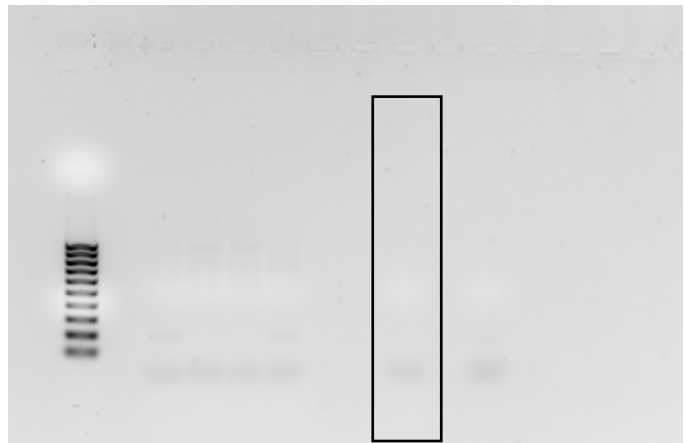

Supplement: Source Data Fig. 3 — Gels from Fig. 3d with ladders. [file 41591_2022_1956_MOESM4_ESM.pdf]
